# Atomic-scale unveiling of multiphase evolution during hydrated Zn-ion insertion in vanadium oxide

**DOI:** 10.1038/s41467-021-24700-w

**Published:** 2021-07-29

**Authors:** Pilgyu Byeon, Youngjae Hong, Hyung Bin Bae, Jaeho Shin, Jang Wook Choi, Sung-Yoon Chung

**Affiliations:** 1 Department of Materials Science and Engineering and KAIST Institute for the Nanocentury, Korea Advanced Institute of Science and Technology, Daejeon, Korea; 2 KAIST Analysis Center, Korea Advanced Institute of Science and Technology, Daejeon, Korea; 3 School of Chemical and Biological Engineering and Institute of Chemical Processes, Seoul National University, Seoul, Korea

**Keywords:** Energy, Solid-state chemistry, Structure of solids and liquids, Batteries, Imaging techniques

## Abstract

An initial crystalline phase can transform into another phases as cations are electrochemically inserted into its lattice. Precise identification of phase evolution at an atomic level during transformation is thus the very first step to comprehensively understand the cation insertion behavior and subsequently achieve much higher storage capacity in rechargeable cells, although it is sometimes challenging. By intensively using atomic-column-resolved scanning transmission electron microscopy, we directly visualize the simultaneous intercalation of both H_2_O and Zn during discharge of Zn ions into a V_2_O_5_ cathode with an aqueous electrolyte. In particular, when further Zn insertion proceeds, multiple intermediate phases, which are not identified by a macroscopic powder diffraction method, are clearly imaged at an atomic scale, showing structurally topotactic correlation between the phases. The findings in this work suggest that smooth multiphase evolution with a low transition barrier is significantly related to the high capacity of oxide cathodes for aqueous rechargeable cells, where the crystal structure of cathode materials after discharge differs from the initial crystalline state in general.

## Abstract (web summary)

The detailed understanding of the structural variations during cycling in cathodes for Zn-ion aqueous rechargeable batteries is still limited. Here, the authors utilize atomic-column-resolved scanning transmission electron microscopy to elucidate multiphase evolution during hydrated Zn-Ion insertion in vanadium oxide.

## Introduction

Although Li-ion batteries have been used as a major rechargeable power source since 1991, safety and long-standing cost issues from the utilization of Li have been routinely raised. This has led researchers to seek alternative intercalation chemistry to Li without using flammable organic electrolytes. Various aqueous batteries operated using earth-abundant redox cations have been suggested over the last two decades in response to this^1–7^. As metallic Zn with a high capacity can be used as an anode together with a water-based nonflammable aqueous solution as an electrolyte, Zn-ion aqueous rechargeable batteries have attracted a surge of attention over the last several years^1,2,7–9^. In particular, it was recently demonstrated that noticeable capacities of more than 300 mAh g^-1^ and thousands of cycles can be achieved in V_2_O_5_ and other V-based oxides as cathode materials in aqueous Zn-ion batteries^2,7,10–23^. Despite these notable electrochemical properties especially for large-scale applications, in-depth understanding of the Zn-intercalation behavior and the origin of high capacities and reversibility in vanadium oxides under an aqueous environment remains seriously lacking, whereas there have been numerous reports focusing on the cycling performance.

Atomic-column-resolved scanning transmission electron microscopy (STEM) with spherical aberration correction has been extensively utilized as a suitable analysis tool over the last decade to probe the local structure and composition variations in intercalated oxides for energy storage^24–32^. In this work, we intensively use STEM in (high-angle) annular dark-field ((HA)ADF) and annular bright-field (ABF) modes^33–42^ to precisely identify both the site occupancy of Zn and H_2_O and the multiphase evolution in V_2_O_5_ during discharge of Zn ions under an aqueous condition. More importantly, as Zn insertion proceeds further, the presence of multiple intermediate topotactic phases, which cannot be identified by macroscopic powder diffraction, is directly reveled at an atomic scale. The remarkable reversible capacity and cyclability of V_2_O_5_ thus appear to have a strong correlation with the topotactically smooth transformation between the charged and discharged phases via the intermediate transient states. The findings in this study suggest that the availability for facile multiphase transitions during charge/discharge may be an important condition for cathode materials with high capacity and reversibility in aqueous rechargeable batteries, where the crystal structure of discharged cathodes comprehensively differs from its initial crystalline state in general.

## Results

### X-ray diffraction analysis

**Fig. 1:**
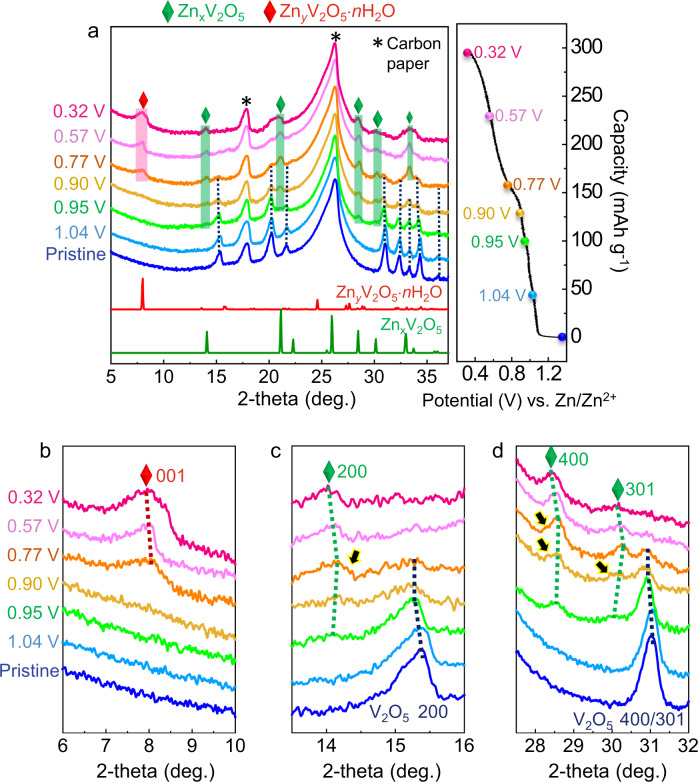
Series X-ray powder diffraction patterns of V_2_O_5_. **a** Each ex situ diffraction pattern was obtained at a different discharge voltage, as indicated in the galvanostatic voltage profile. The generation of two Zn-containing phases (Zn_*x*_V_2_O_5_ and Zn_*y*_V_2_O_5_·*n*H_2_O) during discharge is identified, as denoted by green and red diamonds. Details of the phase identification are provided in Supplementary Figs. 1 and 2. **b**–**d** The narrow-range enlargements are shown for the major Bragg peaks of the discharged phases. Shifts of the peak position during discharge directly indicate the change in lattice parameters and thereby solid-solution behavior. Black arrows denote unidentified small shoulder peaks, implying multiple-phase discharge reactions.

Prior to atomic-scale observation, we carried out an X-ray powder diffraction analysis to examine the phase evolution during discharge. Figure 1a shows a series of diffraction patterns together with a voltage profile during Zn intercalation into V_2_O_5_ particles. Each of the patterns was obtained at a different discharge voltage. This set of results indicates the formation of two new phases during discharge, as denoted by red and green diamonds in the diffraction patterns. Figure 1b−d provide enlargements within 2θ ranges for the Bragg reflections of the new phases. First, left-hand shifts of the V_2_O_5_ (200) and (301) peaks upon discharging are observed (Fig. 1c, d) from the beginning to 0.95 V, directly demonstrating the lattice parameter increment of solid-solution V_2_O_5_ with Zn and thereby a gradual drop in the voltage profile in Fig. 1a. In addition, as a new Zn-intercalation phase, Zn_*x*_V_2_O_5_ (green diamonds), is generated at 0.95 V, the two phases, solid-solution V_2_O_5_ and Zn_*x*_V_2_O_5_, coexist in the voltage range of 0.95–0.77 V (see Supplementary Fig. 1 for details on the phase identification of Zn_*x*_V_2_O_5_ including X-ray diffraction simulations). As another discharged product, a hydrated Zn_*y*_V_2_O_5_·*n*H_2_O (red diamond) phase finally appears at 0.77 V (Fig. 1b), in agreement with the X-ray diffraction results reported in previous studies^10,11^ (see Supplementary Fig. 2 for details on the (001) peak identification of Zn_*y*_V_2_O_5_·*n*H_2_O). It is also intriguing that the Bragg reflections of the discharged phases (Zn_*x*_V_2_O_5_ and Zn_*y*_V_2_O_5_·*n*H_2_O) show a peak shift (green and red broken lines in Fig. 1b–d) during discharge, indicative of solid-solution behavior. More importantly, as denoted by black arrows in Fig. 1c, d, small shoulder peaks around the major Bragg reflections of Zn_*x*_V_2_O_5_ are detectable as well, although their intensity is fairly low. This strongly implies that the discharge reaction may not be simply based on the three distinct phases but rather is associated with multiple-phase complex evolution.

### STEM analyses with thin films

**Fig. 2:**
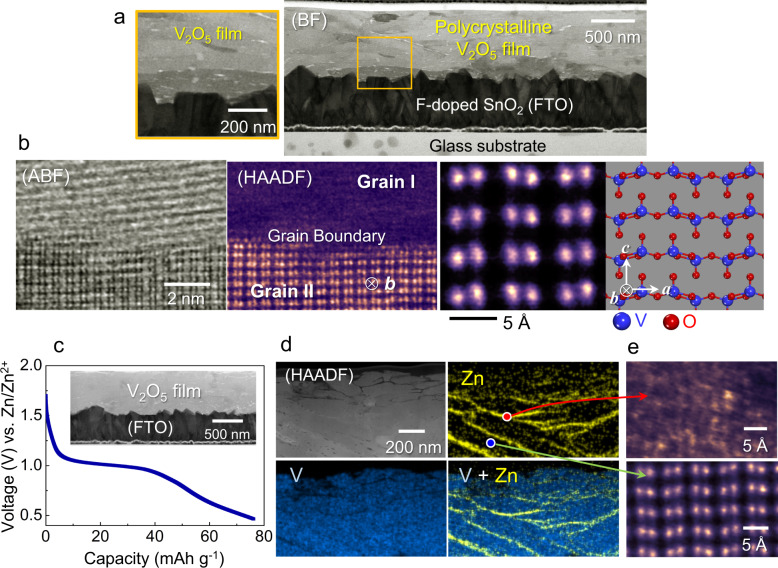
V_2_O_5_ thin-film deposition and electrochemical Zn insertion. **a** The BF-STEM image and its enlargement show the polycrystalline microstructure of the V_2_O_5_ film deposited on a glass substrate coated with F-doped SnO_2_. **b** A grain-boundary region is magnified in a pair of ABF and HAADF images. Grain II is aligned in the [010] projection, as shown in the atomic-column-resolved image along with a schematic illustration for atom positions. **c** A discharge profile demonstrates the electrochemical Zn insertion into the V_2_O_5_ thin film. **d** A set of EDS maps also verifies the presence of Zn, showing a much higher Zn concentration in grain-boundary regions. This sample was discharged to 0.47 V. **e** Two atomic-scale images taken from the grain bulk (lower) and a Zn-rich grain-boundary (upper) are compared to consistently show the low crystallinity of the hydrated Zn_*y*_V_2_O_5_·*n*H_2_O phase.

To scrutinize the phase evolution during discharge, we prepared polycrystalline V_2_O_5_ thin films deposited on a conductive SnO_2_-coated glass substrate instead of using randomly oriented particle-type samples for efficient STEM observation. Figure 2a shows the overall film configuration on a conductive SnO_2_-coated substrate (see Supplementary Fig. 3 for the X-ray diffraction pattern and the chemical composition of the films). As can be seen in the bright-field (BF) STEM image and its enlargement, a polycrystalline microstructure was well developed. When the film was observed at a higher magnification, V_2_O_5_ grains and their boundaries were readily identified. A pair of ABF and HAADF STEM images in Fig. 2b exemplifies two adjacent grains and their boundary, which is a typical crystal−crystal interface with no intergranular phase (see Supplementary Fig. 4 for additional sets of images showing grain boundaries). The lower grain denoted as “Grain II” in the HAADF image is aligned in the [010] projection. Therefore, as shown in the atomic-column image together with the schematic illustration for the atom position in Fig. 2b, the structural feature showing the layered [VO_5_] slabs and interstitial empty sites between the slabs is easily recognized in this *b*-axis projection.

As the conductive SnO_2_ coating on a glass substrate acts as a current collector, Zn ions are electrochemically inserted into each of the grains through grain boundaries in the film. Indeed, galvanostatic charge/discharge profiles could be obtained when the thin-film sample was electrochemically cycled with a 1 M ZnSO_4_/H_2_O electrolyte (see Supplementary Fig. 5a). Figure 2c presents a typical discharge profile acquired from the film at a constant current density. An intriguing aspect during the composition analysis of the film after the discharge reaction is that a notably higher concentration of Zn was identified in the grain-boundary regions, as demonstrated by the energy dispersive X-ray spectroscopy (EDS) maps in Fig. 2d. This compositional information indicates that Zn-ion diffusion along grain boundaries is much faster than the bulk diffusion, as expected in general (see Supplementary Fig. 5b, c for an additional set of EDS maps and line profiles). An X-ray photoemission spectroscopy (XPS) analysis also verifies the presence of Zn as well as the reduction of V by Zn insertion (see Supplementary Fig. 6 for the XPS results). Figure 2e shows an HAADF image (upper) acquired from a Zn-rich grain-boundary region in addition to an image of the V_2_O_5_ bulk grain (lower). As indicated by the X-ray diffraction pattern showing a fairly broad width of the (001) peak (red diamond) at the final stage of discharge in Fig. 1b, a significantly low degree of crystallinity could be consistently identified, representing the hydrated Zn_*y*_V_2_O_5_·*n*H_2_O phase. An additional set of wide-view images for this Zn-rich discharged phase is provided in Supplementary Fig. 7. Although the results shown in Fig. 2d, e were obtained from a thin-film discharged to 0.47 V, a similar Zn distribution and the presence of a low-crystallinity phase in the grain-boundary regions in a film discharged to 0.77 V were observed during the STEM analysis (see Supplementary Figs. 8−11).

### Atomic-column-resolved observations

**Fig. 3:**
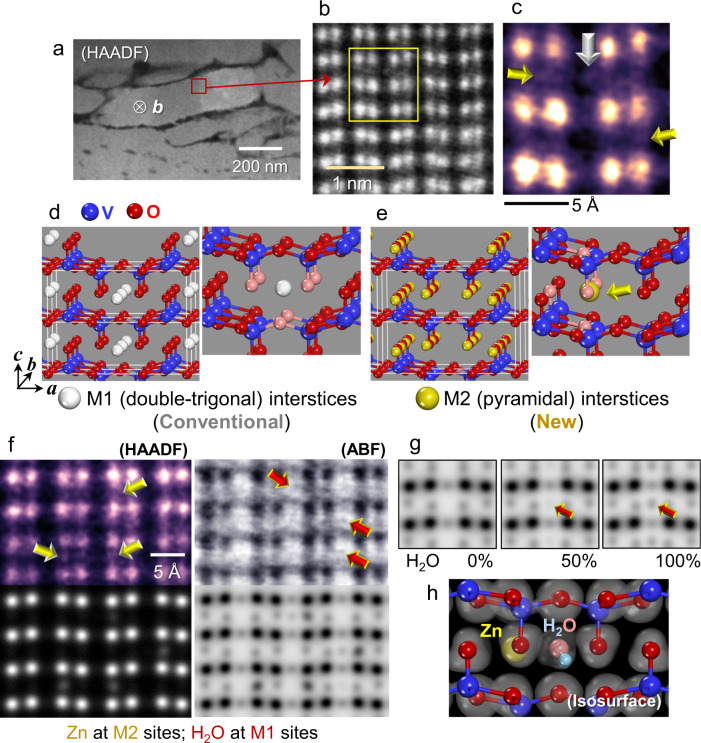
Direct visualization of Zn and H_2_O insertion. **a** The HAADF image shows the polycrystalline microstructure of the discharged film (0.77 V). **b** In addition to the V columns, another bright contrast is identified in this atomic-level image. A yellow rectangle indicates the typical location for the additional contrast between the V columns. **c** A magnified image is provided for the location in **b**. Each of the white and yellow arrows indicates a detectable contrast from the two crystallographically distinct interstitial sites in V_2_O_5_. **d**, **e** The double-trigonal M1 and pyramidal M2 interstices are denoted by white and yellow spheres in the illustrations, respectively. Nearest-neighboring oxygens of each interstice are depicted by light-red spheres. **f** The yellow arrows verify the unconventional Zn occupancy at the M2 sites. While there is no bright contrast at the M1 sites in the HADDF image, a black contrast is clearly observed in the ABF image (red arrows). This reveals H_2_O insertion. The simulated images are in good agreement with the real images. **g** ABF image simulations with H_2_O occupancy demonstrate that visible contrast of H_2_O can be captured when the occupancy is ≥50%. **h** The isosurface contour of electron-density difference is illustrated with a H_2_O molecule as a ligand, demonstrating the stabilization of Zn at the M2 site.

We first observed the center of a grain in the *b*-axis projection, as indicated by the red rectangle in the HAADF image in Fig. 3a. In agreement with Fig. 2b, the atomic-scale HAADF image in Fig. 3b verifies that each of the V columns is straightforwardly resolved in this projection. The most striking feature in Fig. 3b is that many interstitial sites showing a bright contrast by the Zn intercalation differ from the previously known interstitial sites that other cations, such as Li and Mg, occupy. For clarification, an enlargement of the location denoted by a yellow rectangle in Fig. 3b is provided in Fig. 3c. Previous reports on *M*_*x*_V_2_O_5_ (*M* = Li, Mg; *x* ≤ 1), prepared via electrochemical intercalation or chemical syntheses, have demonstrated that the cations, *M*, locate at the double-trigonal (M1) interstitial sites^43–49^, as represented by white spheres in the schematic illustrations in Fig. 3d. In contrast, our observation directly shows that a majority of Zn ions in our sample unusually occupy the pyramidal (M2) interstitial sites (see yellow arrows in Fig. 3c), as represented by yellow spheres in the schematic illustration in Fig. 3e.

Based on the Fourier transform infrared (FTIR) spectroscopy of our thin-film sample after the discharge reaction for Zn intercalation, a substantial absorption peak at a wavelength of ~3200 cm^−1^ was detected (see Supplementary Fig. 12). As it is known that this peak stems from the vibrational absorbance of O−H stretching, the FTIR results indicate the insertion of H_2_O molecules into the lattice during the discharge^17^. We thus simultaneously acquired HAADF and ABF images to directly visualize H_2_O in addition to high atomic-number (Z) cations. As indicated by yellow arrows in the HAADF image in Fig. 3f, additional bright contrast showing Zn intercalation at the pyramidal M2 sites could be verified, in agreement with Fig. 3c. More importantly, while no intensity above the background noise is detected at the double-trigonal M1 sites in the HAADF image, substantial black (or gray) contrast is observed in the ABF image in Fig. 3f, as emphasized by red arrows. The clear M1-site contrast in the ABF image provides direct atomic-scale evidence of the H_2_O insertion.

To verify the intercalation of Zn and H_2_O, a series of image simulations in the HAADF and ABF modes were performed by using the multislice method^50^. As demonstrated in Supplementary Fig. 13, imaging at an under-focus condition in the ABF mode with thin samples (≤5 nm in thickness) failed to visualize the oxygen columns. We thus simultaneously obtained all the HAADF and ABF images in in-focus or slightly over-focus conditions to achieve sufficient contrast. The simulated ABF images in Fig. 3g support that the contrast of H_2_O can be clearly captured when the H_2_O occupation in the M1 sites is ≥50%. The whole series of (HA)ADF and ABF image simulations with the occupation variation of Zn and H_2_O are also provided in Supplementary Figs. 14 and 15, respectively. Good agreement between the experimentally acquired and simulated images is noted in Fig. 3f (see Supplementary Fig. 16 for each of the specific Zn and H_2_O occupation factors adopted in the image simulations), revealing the unusual site occupancy of Zn and the position of H_2_O molecules in V_2_O_5_. In addition, as demonstrated by the electron-density isosurface obtained by ab initio density functional theory (DFT) calculations in Fig. 3h, the H_2_O molecule acts as the nearest ligand and thus constructs a weak bond with Zn. Consequently, the presence of H_2_O as crystal water^17,51,52^ appears to play a critical role in stabilizing the Zn occupancy at the pyramidal M2 site, strongly supporting hydrated Zn-intercalation during discharge. More details on the DFT calculations are included in Supplementary Fig. 17.

**Fig. 4:**
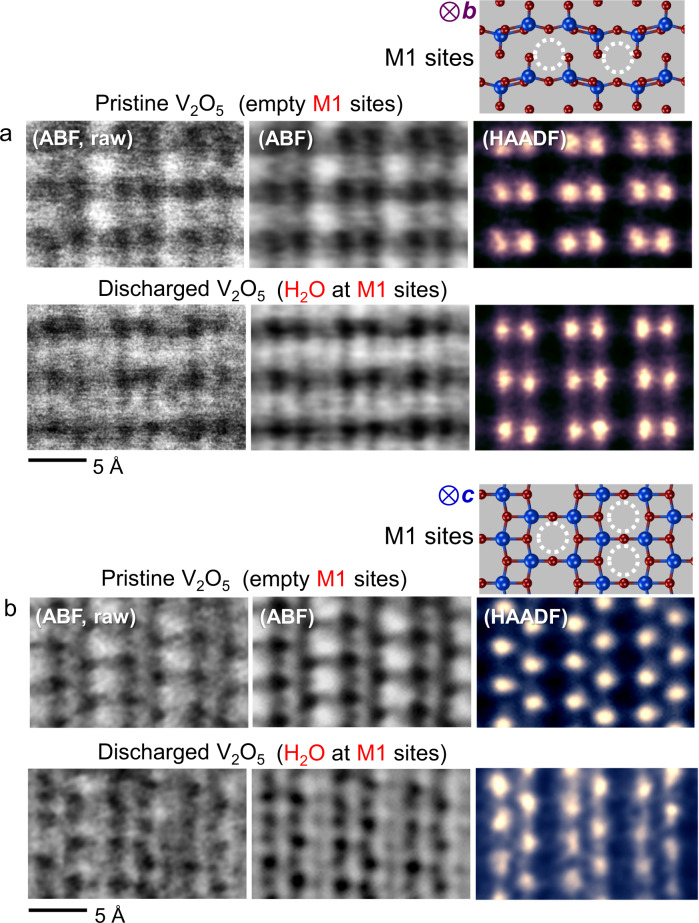
STEM images in two projections to visualize the presence of H_2_O. **a** When neither Zn nor H_2_O intercalates, no additional contrast appears in either the HAADF or ABF image in this projection. A black contrast is clearly observed at the M1 sites in the ABF image of a discharged sample. This reveals the H_2_O insertion at the M1 sites. **b** These sets of images were acquired in the [001] projection. A substantial black contrast in the ABF image of a discharged sample consistently demonstrates the presence of H_2_O at the M1 interstice.

To consolidate our observation of the H_2_O intercalation at the M1 sites, additional sets of simultaneously obtained ABF and HAADF images in both the [010] and the [001] projections are provided in Fig. 4. While the M1 sites are identified to be empty in pristine films in both projections, substantial column contrast is clearly detected at some M1 interstices exclusively in the ABF images, not in the HAADF images. The contrast feature appearing only in ABF mode thus consistently supports the presence of H_2_O with low atomic numbers. Raw ABF images without band-pass filtering are also provided in Fig. 4 to clarify that this image aspect is not affected by filtering. Inverse-intensity ABF images obtained in the [001] projection are shown in Supplementary Fig. 18 for better visualization of the presence of H_2_O.

### Multiple phase transformations

**Fig. 5:**
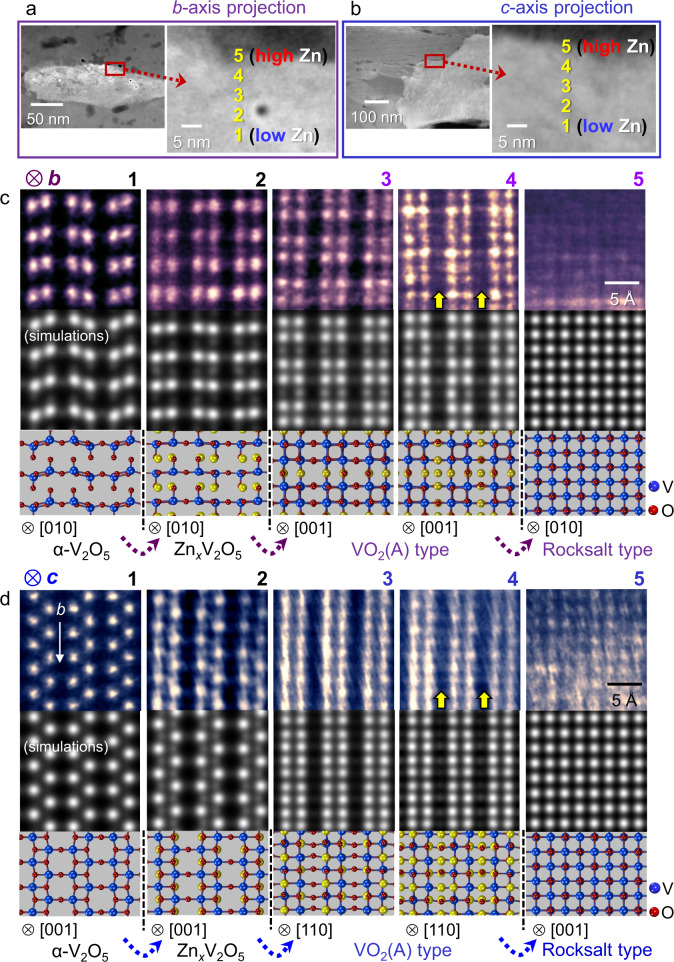
Topotactic multiphase transformations. **a**, **b** This sample was discharged to 0.77 V. Boundary regions of grains in both *b*- and *c*-axis projections were observed, as denoted by a red rectangle in each projection. A larger amount of Zn is detected when the location for observation approaches the grain-boundary. **c**, **d** Magnified ADF images from locations 1−5 and corresponding simulated images are provided in each projection together with schematic illustrations of the atom positions. Yellow arrows indicate the sites showing a detectable intensity by Zn intercalation.

The key finding in this work was acquired when we examined the high Zn-concentration regions near grain boundaries. Figure 5a, b show ADF images of V_2_O_5_ grains in the *b*- and *c*-axis projection, respectively. The magnified regions with a high Zn concentration, as denoted by a red rectangle in each projection, were scrutinized. Two series of atomic-column-resolved ADF images taken from locations 1 to 5 in each grain in Fig. 4c, d directly prove the multiphase transformation at a nanoscale. A detectable intensity is exhibited at many of the atomic columns for the pyramidal interstitial M2 sites in location 2, indicating a substantial amount of intercalated Zn in the Zn_*x*_V_2_O_5_ phase. The most significant observation from Fig. 5 is that the presence of intermediate states (VO_2_(A)-type and rocksalt VO-type structures), which are very difficult to detect by macroscopic powder diffraction, is clearly identifiable at atomic resolution, as revealed in the images for locations 3−5. The structure observed in location 3 in both projections matches well with that of VO_2_(A) among many polymorphisms found in VO_2_ (see Supplementary Fig. 19 for the polymorphisms). Yellow arrows in the images for location 4 denote intercalated Zn in the interstitial sites in the VO_2_(A)-type structure. On the basis of the atomic configuration in the images of locations 3 and 4 in both projections, the crystal structure of locations 3 and 4 appears to be basically an identical VO_2_(A) type, showing that many of the interstitial sites are filled with Zn at location 4 as a solid solution. The rocksalt-type VO structure in both projections is also found in location 5. As reported in a recent study on a vanadium oxide^53^ in addition to other Li-intercalated metal oxides^26,27,30^, the rocksalt structure having cations in every octahedral site in the close-packed oxygen-anion framework appears to be a local minimum^54^ in the overall energy landscape during the transformation of V_2_O_5_, even though the intercalation site of Zn is distinct. A wider view of the rocksalt phase is provided in Supplementary Fig. 20.

To examine how consistently the experimentally acquired images of the intermediate states match the proposed crystal structures, we carried out ADF images simulations for all the images in both projections shown in Fig. 5. As readily recognized, good agreement between the real and simulated images is noted, providing compelling support for the multiple transformation during the discharge reaction. The occupation factor of Zn in the interstitial sites in each structure was properly adjusted so as to show consistent column intensity (see Supplementary Figs. 21 and 22 for the simulated images of the VO_2_(A)-type and rocksalt-type VO structures with Zn at the interstitial sites and Supplementary Fig. 23 for the specific Zn-occupancy factors used for the image simulations in Fig. 5). It is also noted that the transition from V_2_O_5_ to VO_2_(A) can easily take place via simple crystallographic shearing along the [011] direction (see Supplementary Fig. 24 for step-by-step schematic illustrations). Because some of the M2 interstitial sites in the V_2_O_5_ phase are occupied by Zn ions, the V sites in the VO_2_(A) phase after transformation may contain Zn ions. Although we presented the V sites merely with V atoms in the structure illustrations for the VO_2_(A) phase for simplicity (locations 3 and 4), both V and Zn locate in the V sites in the VO_2_(A) and rocksalt VO phases.

**Fig. 6:**
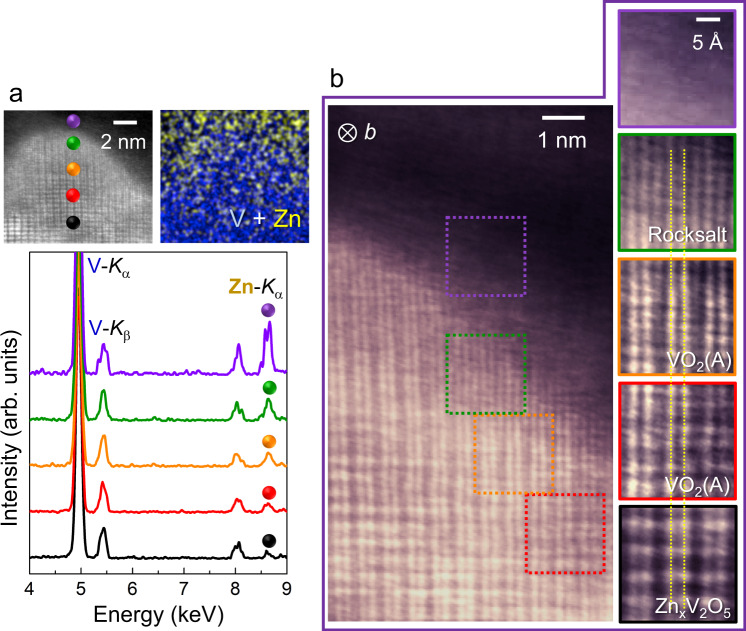
Smooth transformation behavior during Zn insertion in the b projection. **a** As denoted by small spheres in color in the image, gradual increase in Zn concentration near the grain-boundary in a sample discharged to 0.77 V is verified in a series of EDS spectra along with the compositional map. **b** The magnified ADF image and the enlargements for the local regions denoted by squares demonstrate the structurally smooth and continuous phase transition without showing abrupt lattice discontinuity. A pair of yellow lines on the enlargements directly indicates the continuous arrangement of atomic columns in the phases.

**Fig. 7:**
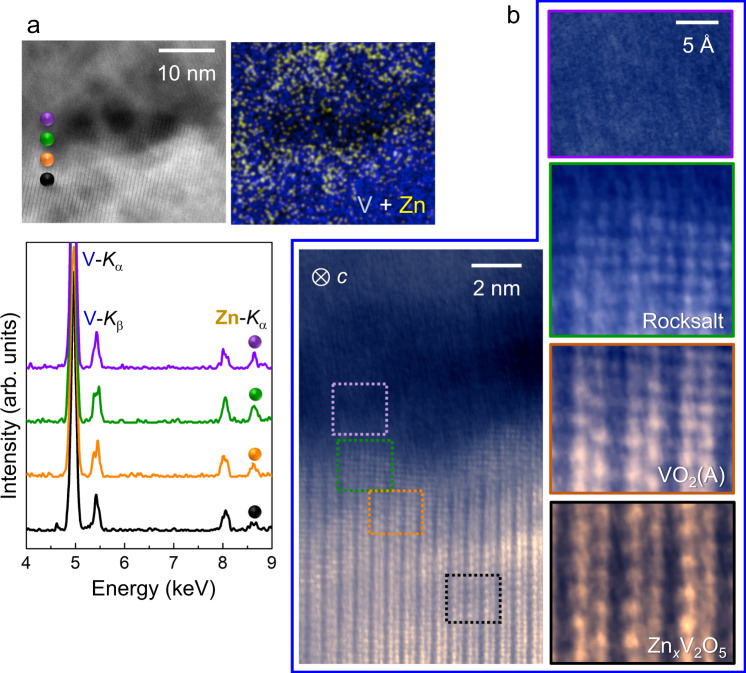
Smooth transformation behavior during Zn insertion in the c projection. STEM images taken in the *c* projection and EDS results are provided to consistently show smooth transformation behavior as well. **a** As denoted by small spheres in color in the image, consistent variation of Zn concentration near the grain-boundary is shown in a series of EDS spectra along with the compositional map. **b** This ADF image and the enlargements for the local regions denoted by squares visualize the continuous phase transition.

Further images were acquired along with chemical verification to clarify the continuous topotactic phase transition behavior with Zn insertion. Figure 6 shows a typical *b*-projection ADF image demonstrating the phase evolution during the discharge. Each enlargement represents the local atomic-column image of a region denoted by a square in color. A continuous and smooth transformation without showing clear-cut phase boundaries is observable in this image. As denoted by a pair of yellow lines in the enlargements, the projected distance between the two adjacent V columns does not significantly vary between the α-V_2_O_5_, Zn_*x*_V_2_O_5_, VO_2_(A), and VO phases. This notable topotactic structural correlation between the four phases is schematically depicted in detail in Supplementary Fig. 25 by comparing the crystal structures in three major orientations. In particular, a series of EDS spectra together with the composition map directly indicate the consistent increment of the Zn concentration with transformation from Zn_*x*_V_2_O_5_ (black) to the low-crystallinity Zn_*y*_V_2_O_5_·*n*H_2_O phase (purple). To confirm the continuous phase transformations with no abrupt structural change, we acquired another ADF image in the *c* projection and enlargements of this image along with EDS spectra and maps, as shown in Fig. 7. Seven additional sets of ADF images in the *b* projection and composition information for the Zn distribution are provided in Supplementary Figs. 26−32, and they consistently support the topotactic transition behavior shown in Fig. 5.

## Discussion

The present study offers several important implications regarding Zn insertion under an aqueous condition in V_2_O_5_. First, our STEM observations together with the DFT calculations provide noteworthy findings showing that many Zn ions unusually intercalate into the pyramidal M2 sites, while H_2_O molecules place at the M1 sites in V_2_O_5_. As the V_2_O_5_ framework with the M2-site Zn occupation is structurally close to VO_2_(A) (see Fig. 5c), this unusual occupancy and the subsequent structural analogy are believed to be energetically beneficial for facile phase transformation with a low transition barrier. The topotactic multiphase transformations observed in this work are thus likely to be responsible for the notably long cycle life behavior in Zn−V_2_O_5_ aqueous cells, as reported in recent studies, despite that the structure of the discharged Zn_*y*_V_2_O_5_·*n*H_2_O phase with low-crystallinity considerably differs from the initial V_2_O_5_ structure. It is worthwhile to mention that proton insertion could be ruled out in our work. As shown in Supplementary Fig. 33, we identified a negligible discharge capacity (<4 mAh g^-1^), when a sulfuric-acid solution of the same pH value was used for comparison. Therefore, the multiphase transformations demonstrated in this study are verified to take place during the intercalation and subsequent conversion reaction of hydrated Zn ions in V_2_O_5_.

We have identified that Zn ions under an aqueous condition unusually intercalate into the pyramidal interstitial sites, in addition to directly visualizing the presence of H_2_O in the V_2_O_5_ lattice. Moreover, as Zn insertion proceeds further, the presence of VO_2_(A)- and VO-rocksalt-type intermediate phases was unveiled at atomic resolution together with the appearance of a hydrated phase for the final discharge product, showing a remarkable topotactic analogy with the intercalated V_2_O_5_. Our work suggests that the availability of facile smooth phase transformations via intermediate states during charge/discharge may be an important condition to retain significantly high capacity and notable cyclability in oxide-based cathodes in aqueous Zn-ion rechargeable batteries, where many cathode materials undergo substantial structure transitions during cycling with aqueous electrolytes.

## Methods

### Thin-film deposition and powder synthesis

Polycrystalline V_2_O_5_ thin films were fabricated on glass substrates with a conductive F-doped SnO_2_ (FTO) coating via a simple electrodeposition process. VOSO_4_·nH_2_O (Alfa Aesar, 99.9%) and a mixture of high-purity ethanol and deionized water (50:50 by vol%) were used to prepare the 0.5 M electrolyte solution for the deposition. The film fabrication was carried out at room temperature in the solution by applying a constant potential of 2 V versus a Ag/AgCl reference electrode with a potentiostat (SP-300, BioLogic). To obtain films with 500−700 nm final thickness, the duration time of the potential application was adjusted to be 2−5 min. After deposition, the film samples were annealed at 300 °C for 12 h in air to acquire crystalline α-V_2_O_5_. Nanocrystalline V_2_O_5_ powder was also used to track the phase evolution by ex situ X-ray diffraction during the discharge reaction. To obtain nanocrystals, a simple dissolution-and- reprecipitation method was utilized. Micron-sized V_2_O_5_ (99.6%, Alfa Aesar) was first dissolved in a 0.3 M oxalic acid solution. This solution was stirred at 80 °C for 5 h for complete dissolution of the V_2_O_5_ particles and subsequently dried at 100 °C to obtain a precursor. Nanocrystalline powders were easily produced by annealing this precursor at 350 °C for 5 h in air. The phase of the grown films and the powder was verified by X-ray diffractometry (Ultima IV, Rigaku) with Cu *K*_α_ radiation.

### Electrochemical reactions

Electrode samples were prepared by casting a slurry of V_2_O_5_ nanocrystals (70 wt%), acetylene black (20 wt%), and polyvinylidene fluoride (PVDF, 10 wt%) with *N*-methylpyrrolidone (NMP) as the solvent on carbon paper. Typical loadings for the electrode coatings were 0.8−1 mg cm^-2^ of the cathode powder. 1 M ZnSO_4_·7H_2_O (99%, Sigma-Aldrich) dissolved in deionized water (pH:4.2–4.8) was employed for Zn insertion and extraction for the aqueous condition. Galvanostatic charge and discharge were carried out with a galvanostat (SP-300, BioLogic) under a constant current density of 0.1 mA cm^-2^ for the thin-film samples and 29.4 mA g^-1^ (0.1 C rate) for the powder samples in a three-electrode beaker cell consisting of a Ag/AgCl (3 M KCl) reference electrode along with a Pt-wire counter electrode. To avoid water splitting during the charge and discharge reactions, the potential range was set to be between −0.5 V and 0.7 V versus the Ag/AgCl reference electrode. As the film thickness could be directly measured through STEM observation, the capacity in the unit of mAh g^-1^ was estimated by assuming that a deposited film is fully dense. Thin-film samples after the second electrochemical cycle were used for STEM observation.

### STEM, EDS, and image simulations

Samples for STEM observation were prepared by lift-out via ion-beam milling in a focused ion-beam system (Helios G4 UX, Thermo Fisher Scientific). Protective amorphous carbon and thin Pt layers were applied over the region of interest before milling. To minimize the sidewall damage and sufficiently thin the specimen for electron transparency, final milling was carried out at a voltage of ~2 kV. STEM images were acquired with a transmission electron microscope (Titan cubed G2 60–300, Thermo Fisher Scientific) at 200 kV with a spherical aberration (Cs) corrector (CEOS GmbH). The images of the discharged films were obtained from samples after the second electrochemical cycle. The optimum size of the electron probe was ~1 Å with a convergence semiangle of 19 mrad. The collection semiangles of the STEM detectors were set to 79.5−200 mrad for HAADF imaging, 19.1−79.5 for ADF imaging, 10.1−19.1 mrad for ABF imaging, and 0−43.3 for BF imaging. To avoid serious specimen damage and obtain reliable images, the beam current was adjusted to be 50−70 pA, the electron dose of which corresponds to 1.4−2.0 × 10^5^ electrons Å^-2^ in our acquisition conditions. As a result, atomic columns could be sufficiently resolved in images unless several scans of an e-beam are repeated to the region of interest (see Supplementary Fig. 34). To reduce background noise and enhance the signal-to-noise ratio in STEM images^55–60^, the obtained raw images were filtered by using the average background subtraction filtering (ABSF) method (https://www.felmi-zfe.at/dm_script/hrtem-filter/). Chemical mapping with EDS was carried out in the Titan cubed G2 at 200 kV along with four integrated silicon-drift EDS detectors at a collection solid angle of 0.7 srad. V-*K*_α_ (4.9 keV) and Zn-*K*_α_ (8.6 keV) lines were selected during elemental mapping. The probe current was adjusted to be 50−100 pA with a scanning time of <250 sec. The EDS maps were low-pass filtered using Bruker ESPRIT software after reduction of background noise for better visualization. STEM images were simulated by using the Dr. Probe software^50^ based on the multislice algorithm. A beam energy of 200 keV, spherical aberration coefficients of *Cs* = 0 mm, *C*_5_ = 0 mm, and *C*_7_ = 0 mm without coma and astigmatism, an electron probe size (FWHM) of 1 Å, and a slice thickness of 2 Å were set during the simulations. Unless specifically mentioned, the simulations were carried out for specimen thickness of 3 nm. Each of the real collection semiangles of the STEM detectors was also used for precise comparison with experimentally acquired images.

### FTIR, XPS, and X-ray diffraction

FTIR spectra to examine the presence of water molecules in the thin films after the Zn insertion reaction were obtained with a FTIR spectrometer (Nicolet 6700, Thermo Fisher Scientific). The samples were dried in an oven at 60 °C for 3 h before the spectroscopy. The valence state variation of V with Zn intercalation was investigated using an X-ray photoelectron spectroscope (K-Alpha XPS, Thermo Scientific) with monochromatic Al-*K*_α_ radiation and flood gun emission of 200 μA. REFLEX (Biobia Inc.) was utilized to simulate the powder X-ray patterns with Cu *K*_α_ radiation. The parameters of the Pseudo Voigt peak shape function were set as *U* = 0.01, *V* = −0.01, and *W* = 0.01 during simulations to obtain a relatively sharp shape for clear discrimination of Bragg peaks.

### DFT calculations

Ab initio DFT calculations for comparison of relative lattice energy values between supercells were carried out using the spin-polarized generalized-gradient approximation (GGA) along with the PBEsol functional revised for exchange correlation of densely packed solids and the ultrasoft pseudopotentials for ionic cores, as implemented in the CASTEP code (Biovia Inc.). To consider energetically favorable configurations of multiple pairs of a Zn ion and a H_2_O molecule in the V_2_O_5_ lattice, sufficiently large 1*a* × 7*b* × 3*c* supercells were constructed for geometry optimization. In addition, the GGA + *U* method with the Hubbard *U* parameter (4.0 eV for V 3*d* states^61^ and 4.7 eV for Zn 3*d* states^62,63^) was employed to account for the electron localization around V and Zn ions. Bases on our convergence testing, we found that a fairly large cutoff energy was required for precise calculations, notwithstanding comparatively high computational cost. The plane-wave basis set for the kinetic energy cutoff was thus set to be 750 eV. Relaxation of the internal coordinates for each atom was performed using the Broyden–Fletcher–Goldfarb–Shanno (BFGS) algorithm with convergence tolerances of 0.1 eV Å^-1^ for the maximum ionic force, 5 × 10^−5^ eV atom^-1^ for the total energy, and 0.005 Å for the maximum ionic displacement.

## Supplementary information

Supplementary Information

## Data Availability

The data that support the findings of this study are available from the corresponding author (S.-Y.C.) upon reasonable request.
