# Supplementary material for: Atomic-scale unveiling of multiphase evolution during hydrated Zn-ion insertion in vanadium oxide
Source: Nat Commun. 2021 Jul 29;12:4599. doi: 10.1038/s41467-021-24700-w (PMC8322084; doi:10.1038/s41467-021-24700-w)
Supplement: Supplementary file 1 — Supplementary Information [file 41467_2021_24700_MOESM1_ESM.pdf]

Supplementary Information

Atomic-Scale Unveiling of Multiphase Evolution during Hydrated Zn-Ion Insertion in Vanadium Oxide

Pilgyu Byeon[1], Youngjae Hong[1], Hyung Bin Bae[2], Jaeho Shin[3], Jang Wook Choi[3*], and Sung-Yoon Chung[1*]

[1]Department of Materials Science and Engineering and KAIST Institute for the Nanocentury, Korea Advanced Institute of Science and Technology, Yuseong-gu, Daejeon 34141, Korea. [2]KAIST Analysis Center, Korea Advanced Institute of Science and Technology, Yuseong-gu, Daejeon 34141, Korea. [3]School of Chemical and Biological Engineering and Institute of Chemical Processes, Seoul National University, Gwanak-gu, Seoul 08826, Korea.

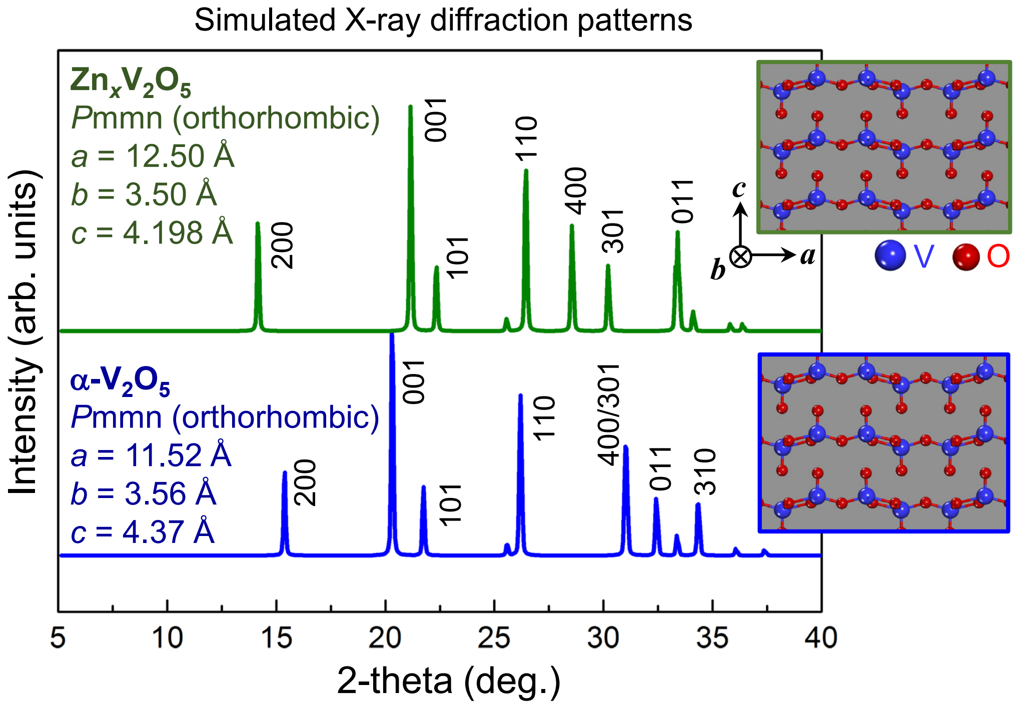

Supplementary Fig. 1 Simulated X-ray diffraction patterns. The first simulated diffraction pattern (green) for $Zn_xV_2O_5$ was obtained by using a unit cell, which has the same fractional atom position and space group of those of α-V_2O_5 but different lattice parameters. As shown in Figure 1a in the main text, the position of the Bragg peaks from the first appearing discharged phase (green diamonds) matches very well with the peak positions of this simulated pattern. The blue simulated pattern for α-V_2O_5 is also provided to clarify the variation of peak positions by the change of lattice parameters.

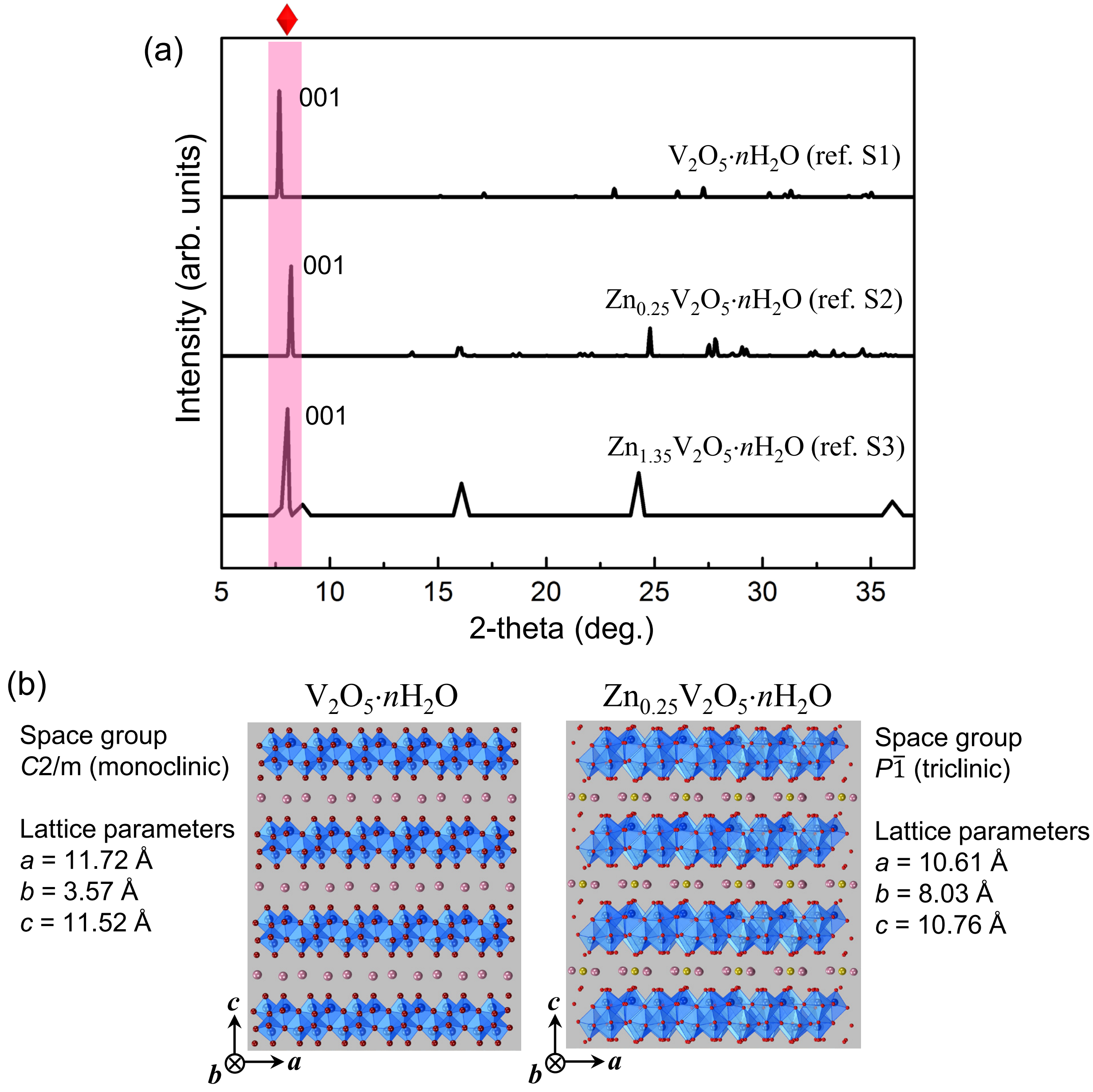

Supplementary Fig. 2 X-ray diffraction patterns and structures between hydrated vanadium oxides. (**a**) The diffraction peaks are adopted from Supplementary Ref. S5–S7. As denoted by a red shadow with a diamond, the major (001) peaks of three hydrated $Zn_yV_2O_5 \cdot nH_2O$ forms are located in very close proximity between 7.5° and 8.5° in the 2θ range. This directly indicates that the *c*-axis lattice parameter of these hydrated phases is hardly affected by the concentration of Zn (ref. S1. Petkov, V. et al. *J. Am. Chem. Soc.* **124**, 10157 (2002); ref. S2. Oka, Y. et al. *J. Solid State Chem.* **126**, 65 (1996); ref. S3. Kundu, D. et al. *Nat. Energy* **1**, 16119 (2016)). (**b**) The crystal structures of $V_2O_5 \cdot nH_2O$ and $Zn_{0.25}V_2O_5 \cdot nH_2O$ are exemplified to show that the presence of interlayer crystal water between the [VO_x] polyhedra is as a common feature in $Zn_yV_2O_5 \cdot nH_2O$ forms. Based on this layered structural characteristic, the interlayer distance and the subsequent position of the (001) peak in $Zn_yV_2O_5 \cdot nH_2O$ forms appear to be influenced by the crystal water with a comparatively large molecular volume rather than much smaller Zn^{2+} cations. In Figure 1a in the main text, the diffraction pattern of $Zn_{0.25}V_2O_5 \cdot nH_2O$ is provided for representative $Zn_yV_2O_5 \cdot nH_2O$.

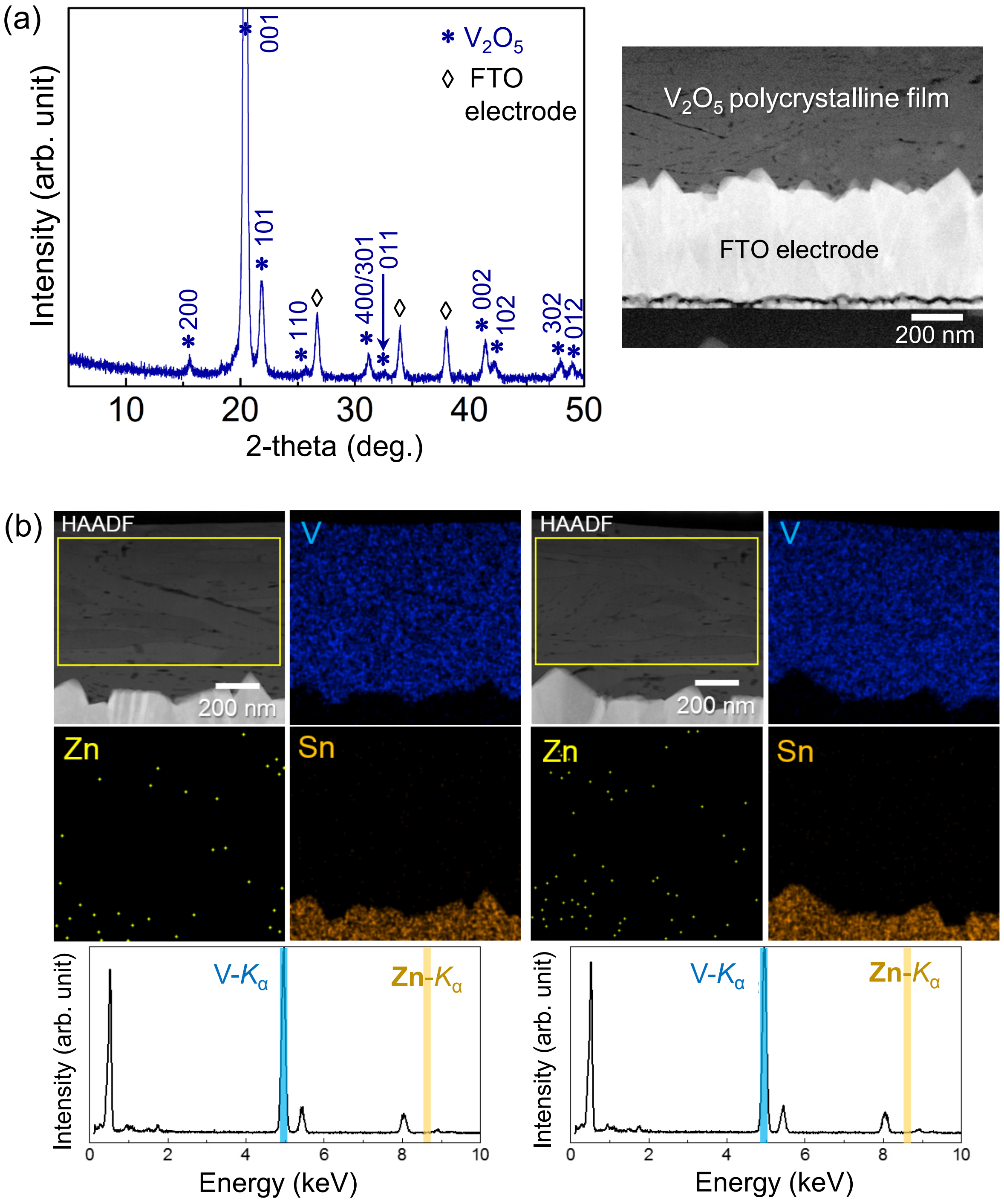

Supplementary Fig. 3 X-ray diffraction and EDS maps for phase identification. (**a**) The X-ray diffraction shows that the deposited pristine thin film is α-V_2O_5, as indicated by asterisks on the diffraction pattern. The HAADF-STEM image also verifies the polycrystalline nature of the film. (**b**) The EDS-based composition analysis verifies the absence of Zn in the thin films. Each EDS spectrum was obtained from the region denoted by a yellow rectangle.

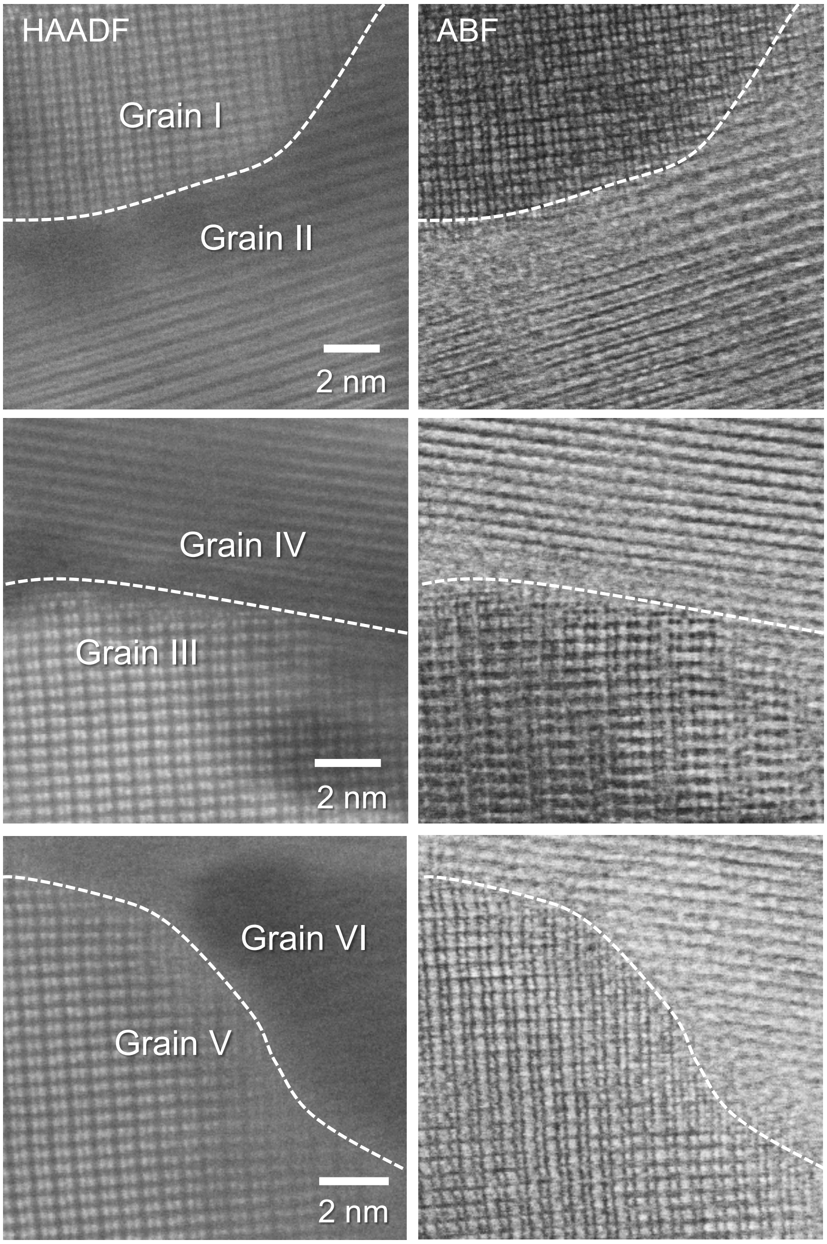

Supplementary Fig. 4 Grain boundaries in pristine V_2O_5 films. Three additional pairs of HAADF and ABF images are provided to verify that grain boundaries in the pristine films are typical crystal–crystal internal interfaces in the polycrystalline microstructure.

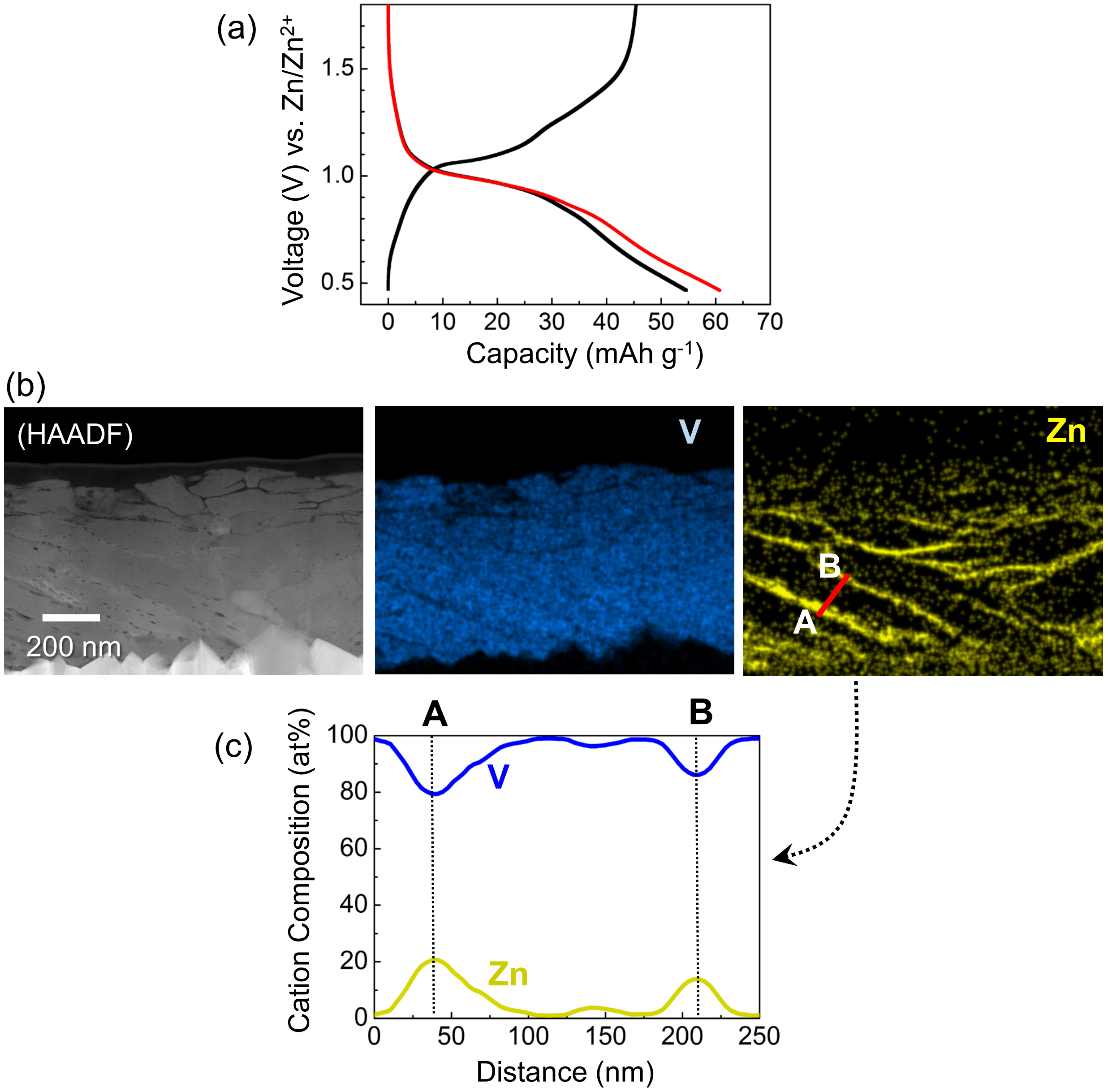

Supplementary Fig. 5 Electrochemical Zn insertion in the film and EDS maps. (**a**) Galvanostatic voltage profiles are provided to show the electrochemical Zn insertion. A pair of black curves indicates the first discharge and charge profiles and the red curves correspond to the second discharge profile of a thin film. (**b**) After the second discharge, EDS maps were obtained to examine the Zn distribution in the thin-film sample. Much higher Zn concentration in the grain-boundary regions is visualized in the composition maps. (**c**) As denoted by a red line on the Zn map in (**b**), line profiles for V and Zn between locations A and B are presented to provide quantitative composition information.

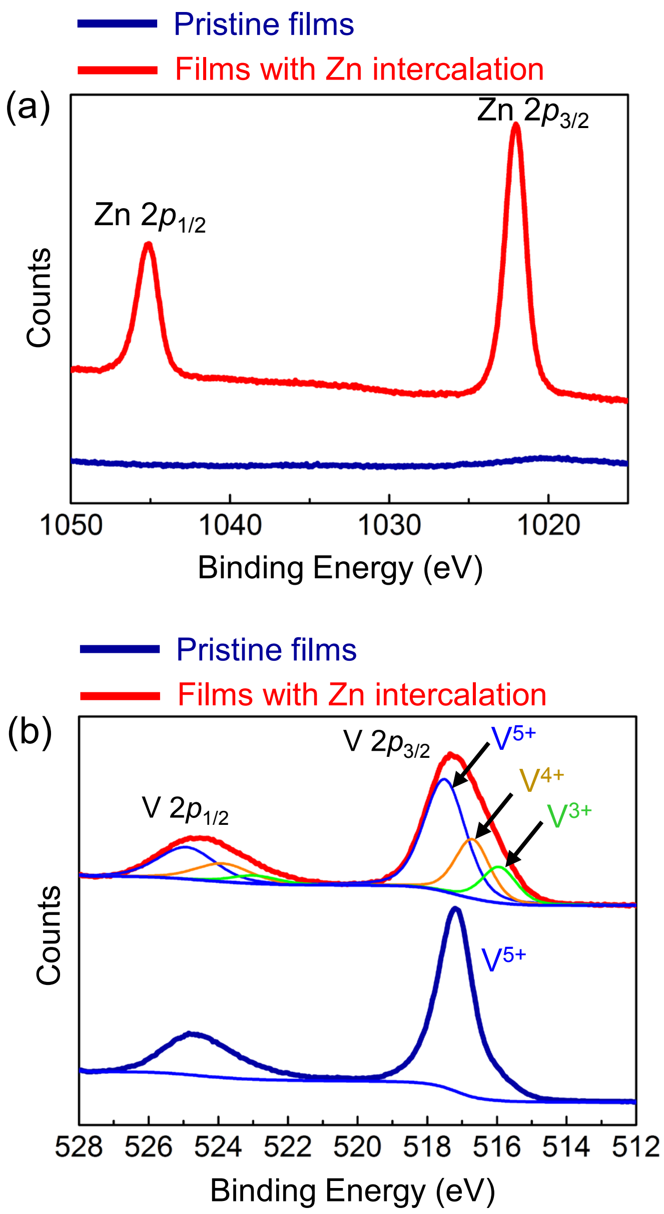

Supplementary Fig. 6 Comparison of XPS. (**a**) The peaks for Zn 2*p* are clearly detected for the sample surface after the discharge reaction, consistently showing the presence of Zn. (**b**) As indicated by the peak deconvolution for the different valence states of V, it is noted that the V $2p_{3/2}$ peak is much wider after the discharge reaction. Therefore, the reduction of V^{5+} by the Zn intercalation is apparent from the XPS analysis.

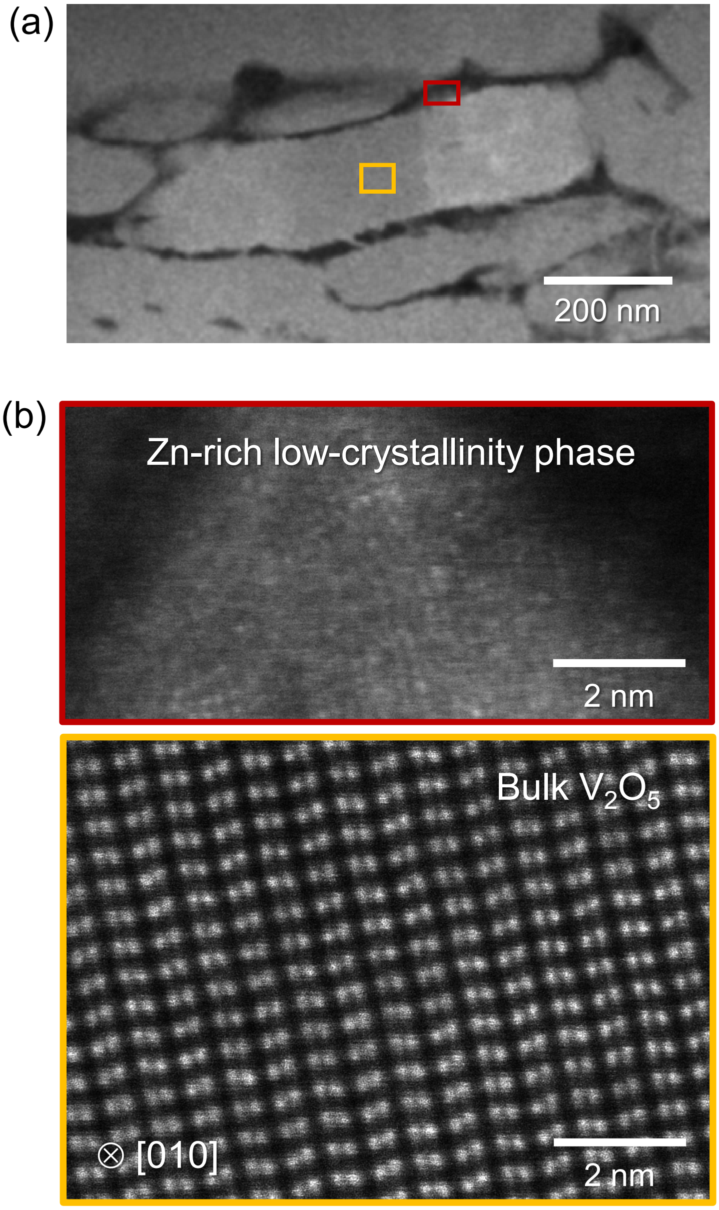

Supplementary Fig. 7 Additional STEM images for a discharged phase. (**a**) This shows the overall polycrystalline microstructure after the discharge reaction for Zn insertion. (**b**) A Zn-rich local phase with low crystallinity is identified from a grain-boundary region denoted by a red rectangle in (a), whereas the central region (orange rectangle) with a low Zn concentration in the grain exhibits preservation of the orthorhombic V_2O_5 phase. Based on our X-ray diffractions shown in Figure 1 as well as the results in previous reports, this Zn-rich low-crystallinity phase is $Zn_yV_2O_5{\cdot}nH_2O$.

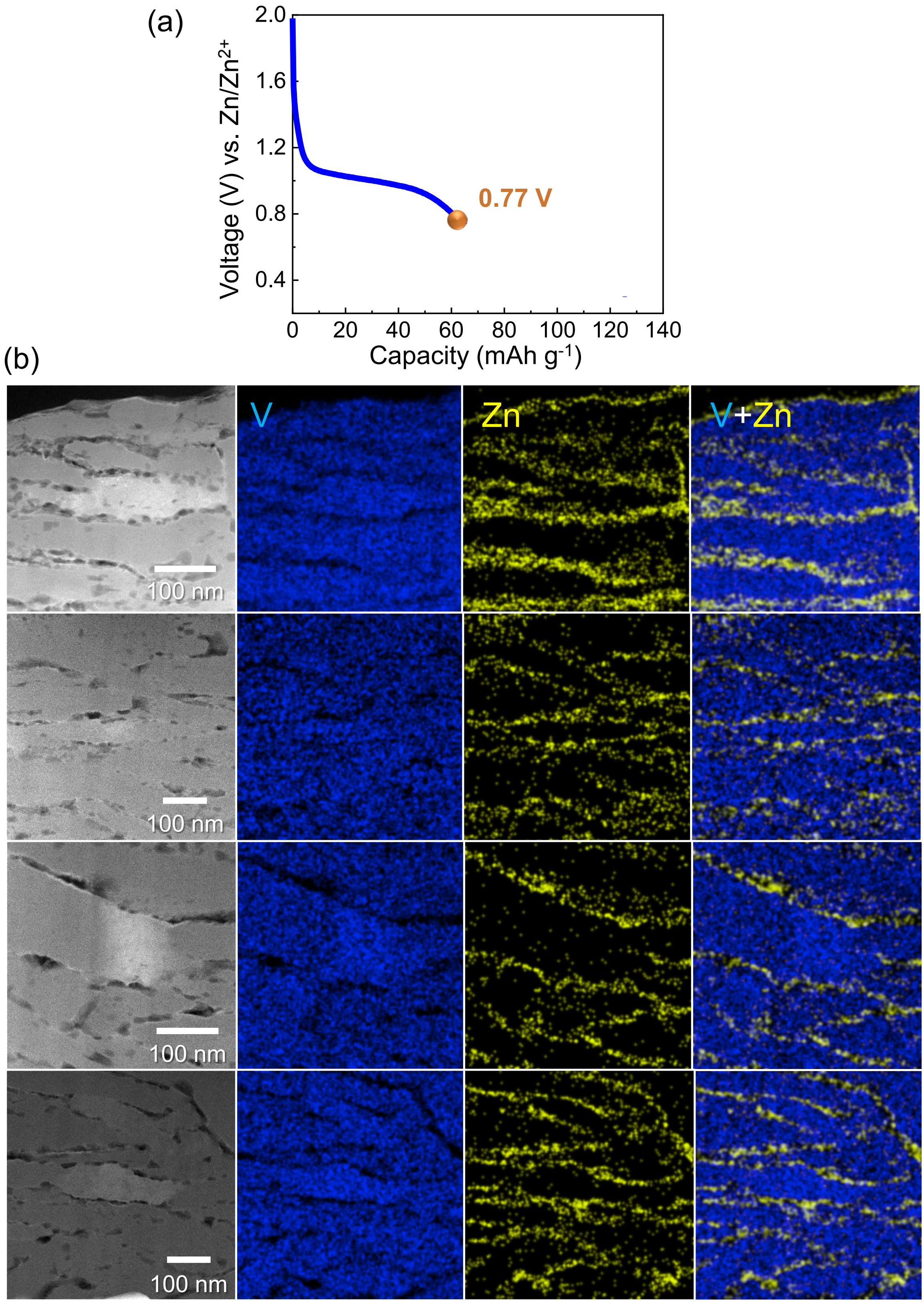

Supplementary Fig. 8 Additional EDS maps of a discharged film sample. (**a**) A galvanostatic voltage profile is provided to show that the discharge cutoff voltage is 0.77 V. (**b**) These additional four sets of EDS maps demonstrate similar Zn distributions in the grain-boundary regions in a film discharged to 0.77 V, although the discharge cutoff voltage is higher than that in Figure 2d in the main text.

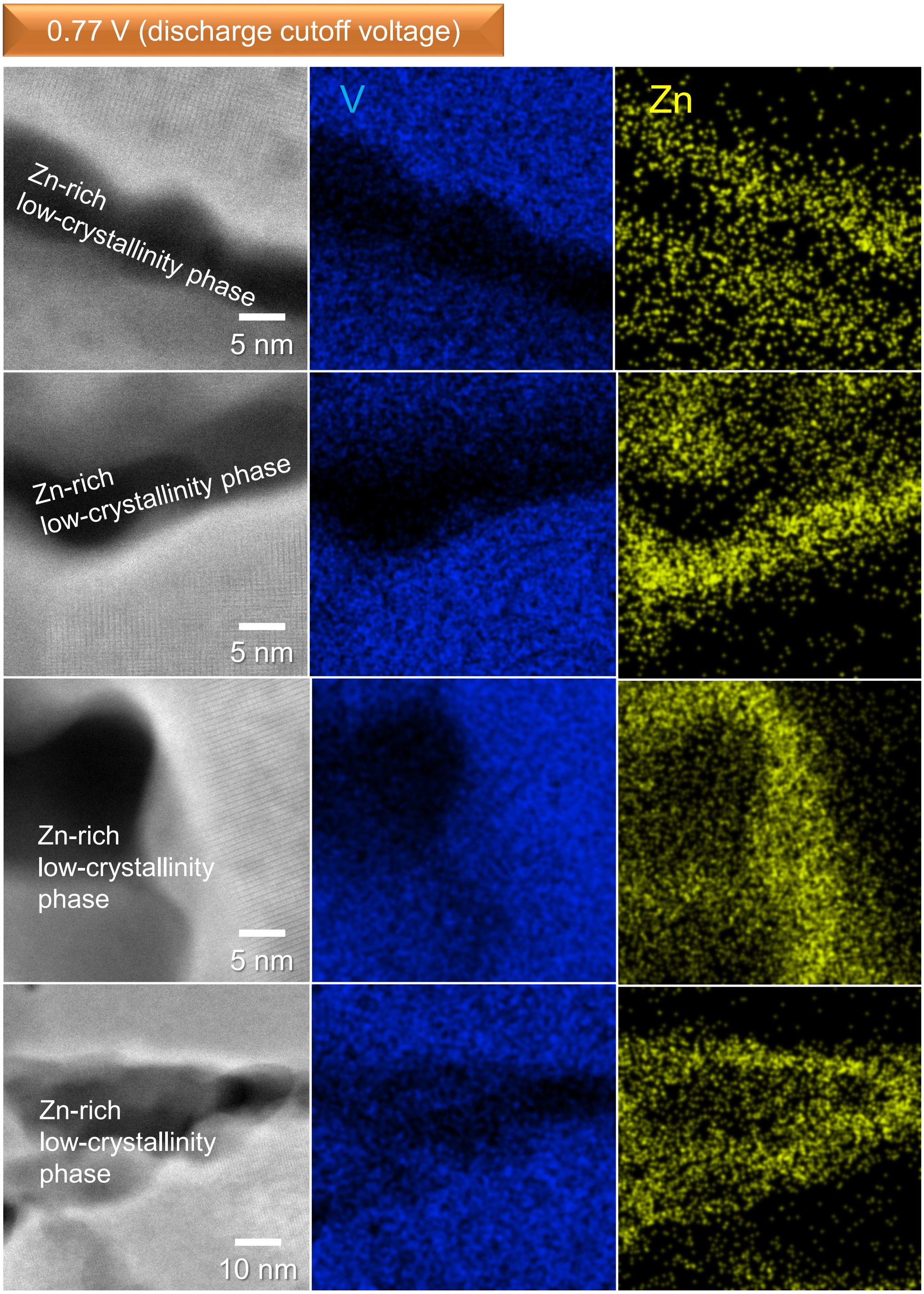

Supplementary Fig. 9 Additional HAADF-STEM images and EDS maps for grain-boundary regions in a discharged sample. The presence of a Zn-rich low-crystallinity phase is verified in the grain boundary regions at a higher magnification.

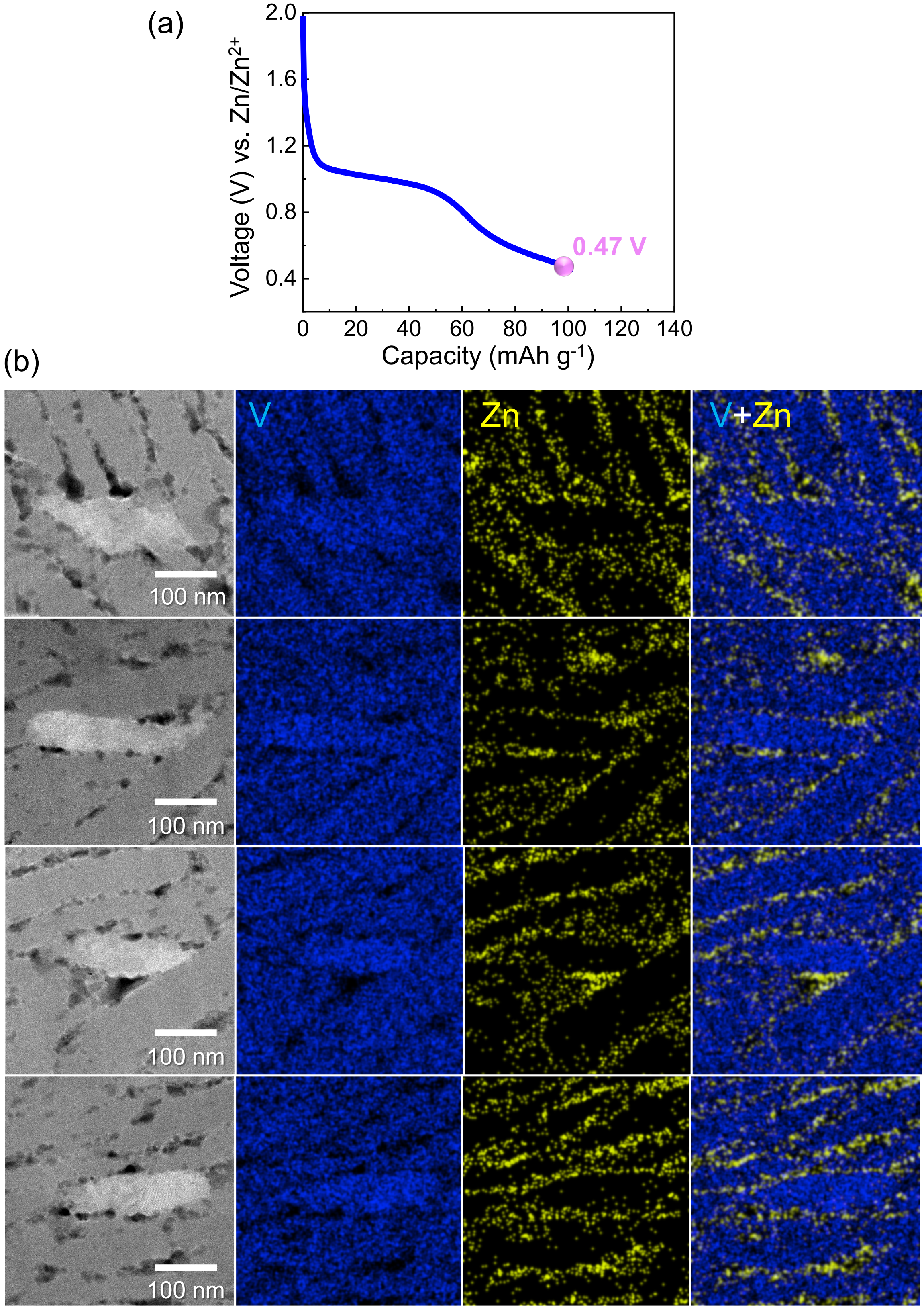

Supplementary Fig. 10 Additional EDS maps of a discharged film sample. (**a**) A galvanostatic voltage profile is provided to show that the discharge cutoff voltage is 0.47 V. (**b**) These additional four sets of EDS maps confirm the consistent Zn distribution in the grain-boundary regions in a film discharged to 0.47 V, as already shown in Figure 2d in the main text.

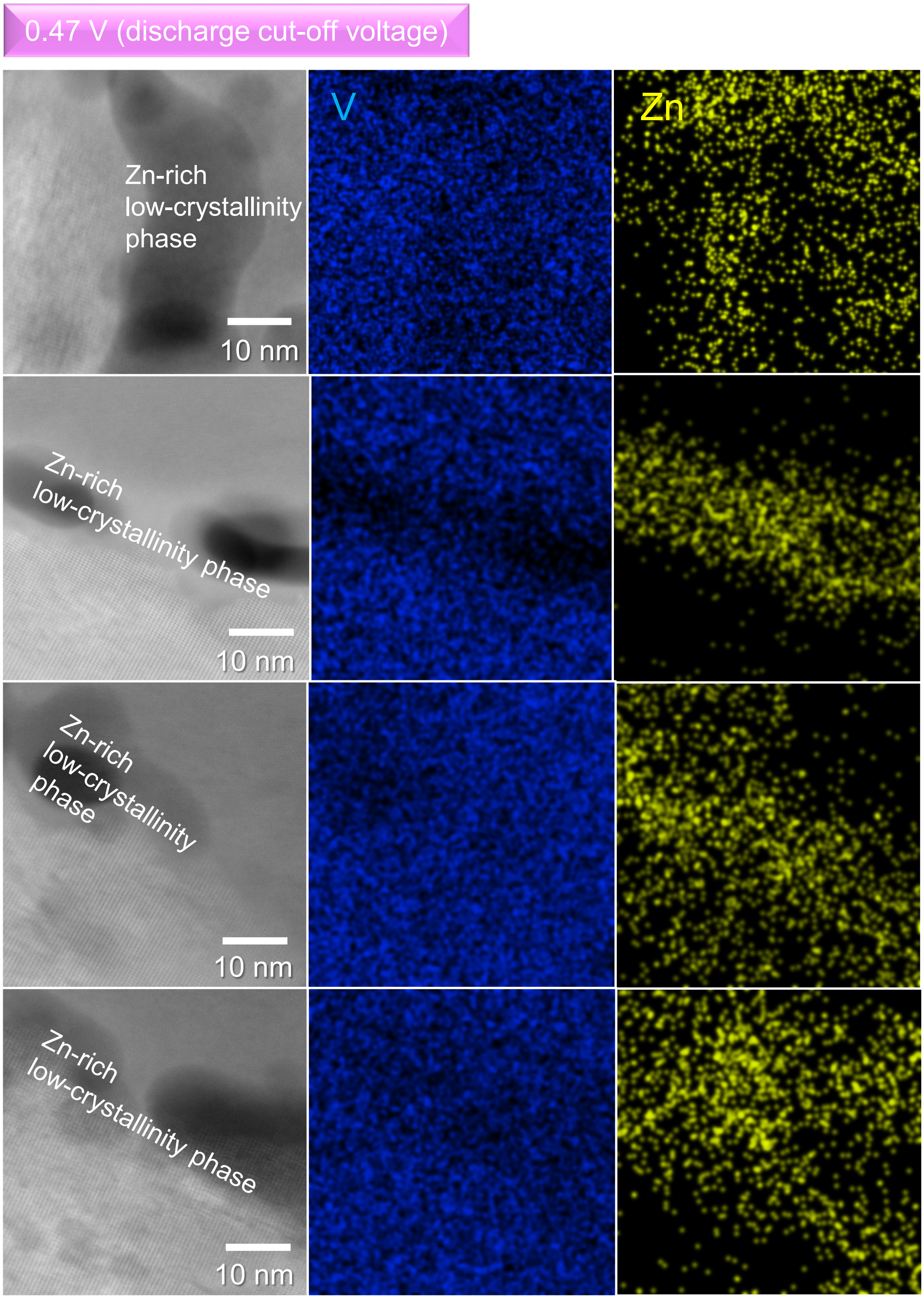

Supplementary Fig. 11 Additional HAADF-STEM images and EDS maps for grain-boundary regions in a discharged sample. The presence of a Zn-rich low-crystallinity phase is verified in the grain boundary regions at a higher magnification. The thickness of the phase appears to increase in this sample discharged to 0.47 V.

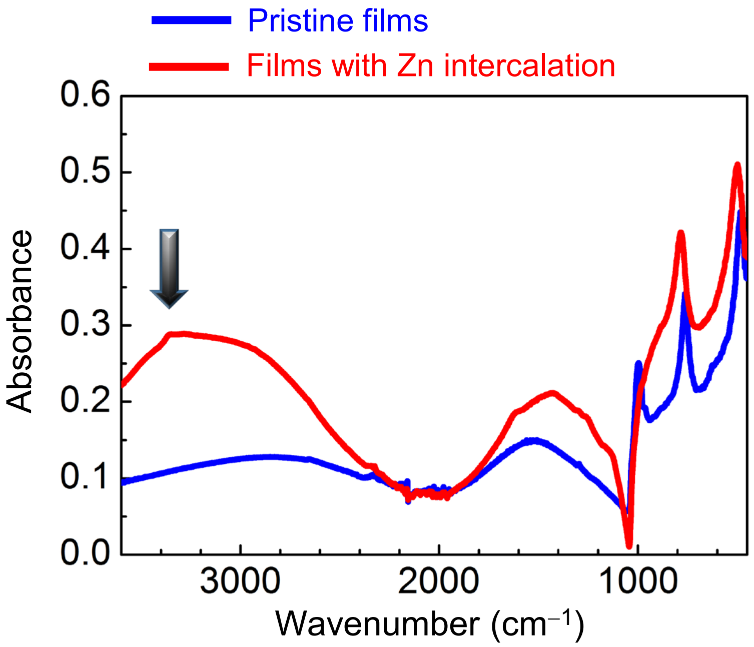

Supplementary Fig. 12 Fourier transform infrared (FTIR) spectroscopy. As denoted by an arrow, a strong absorbance peak appears at a wavelength of ~3200 cm^{-1} after the discharge reaction for Zn intercalation. As this peak indicates the absorption by the O–H stretching, the FTIR analysis supports H_2O co-intercalation with Zn during the discharge.

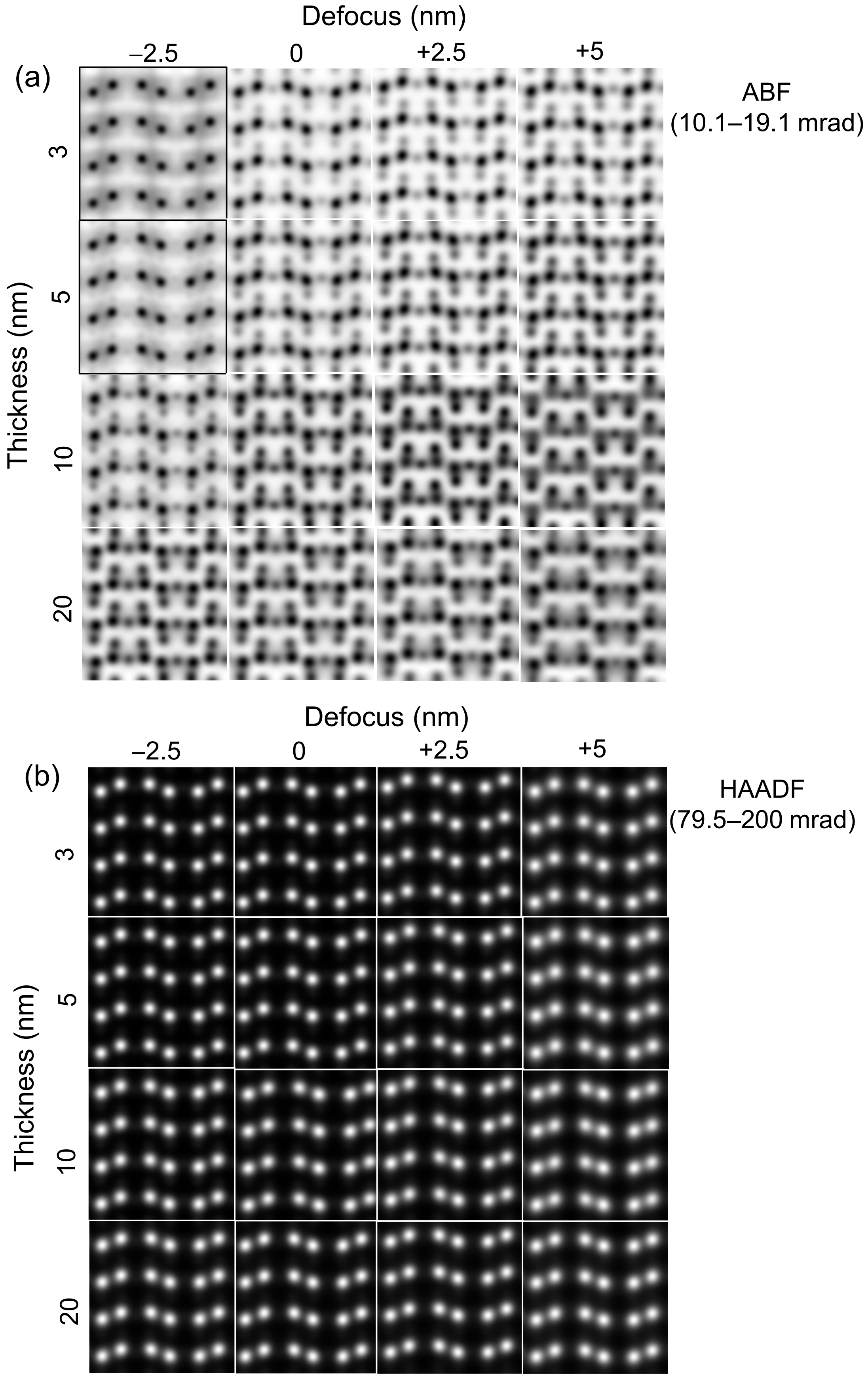

Supplementary Fig. 13 STEM image simulations of V_2O_5 in the *b* projection. (**a**) Imaging at a under-focus condition in the ABF mode with thin samples (≤5nm in thickness) fails to clearly visualize the oxygen columns, as framed by black squares. (**b**) No serious variation in column contrast with different defocus conditions is found in the HAADF mode.

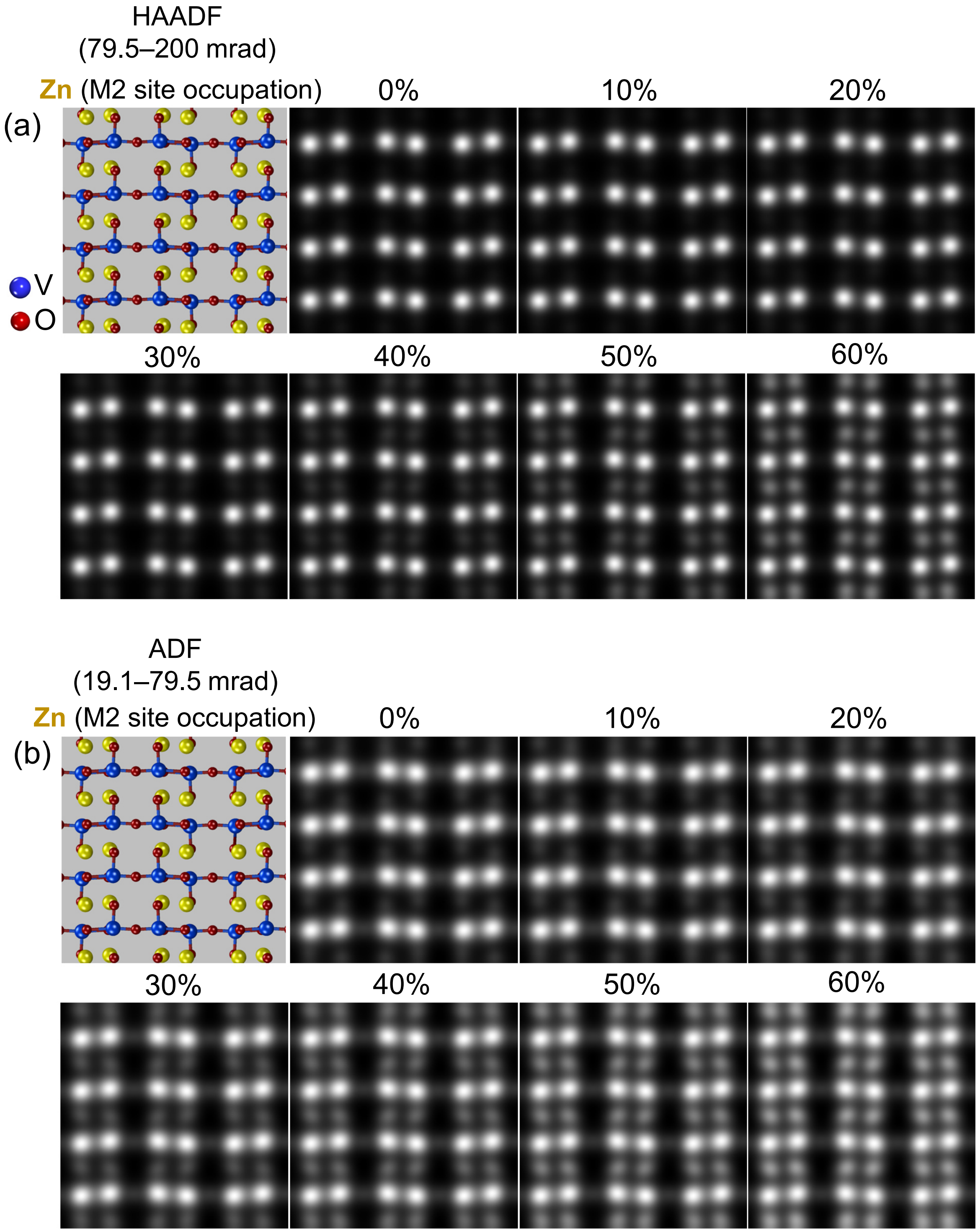

Supplementary Fig. 14 STEM image simulations with Zn occupancy at the M2 sites. (**a**) The proportional increment of the M2-site column intensity to the Zn occupancy factor is demonstrated in the HAADF mode. (**b**) As the collection semiangle in this ADF mode is lower than that of the HAAADF mode, the column contrast at the M2 sites by the Zn occupation is more sensitively detectable, showing a sufficient intensity even at 20% occupancy by Zn.

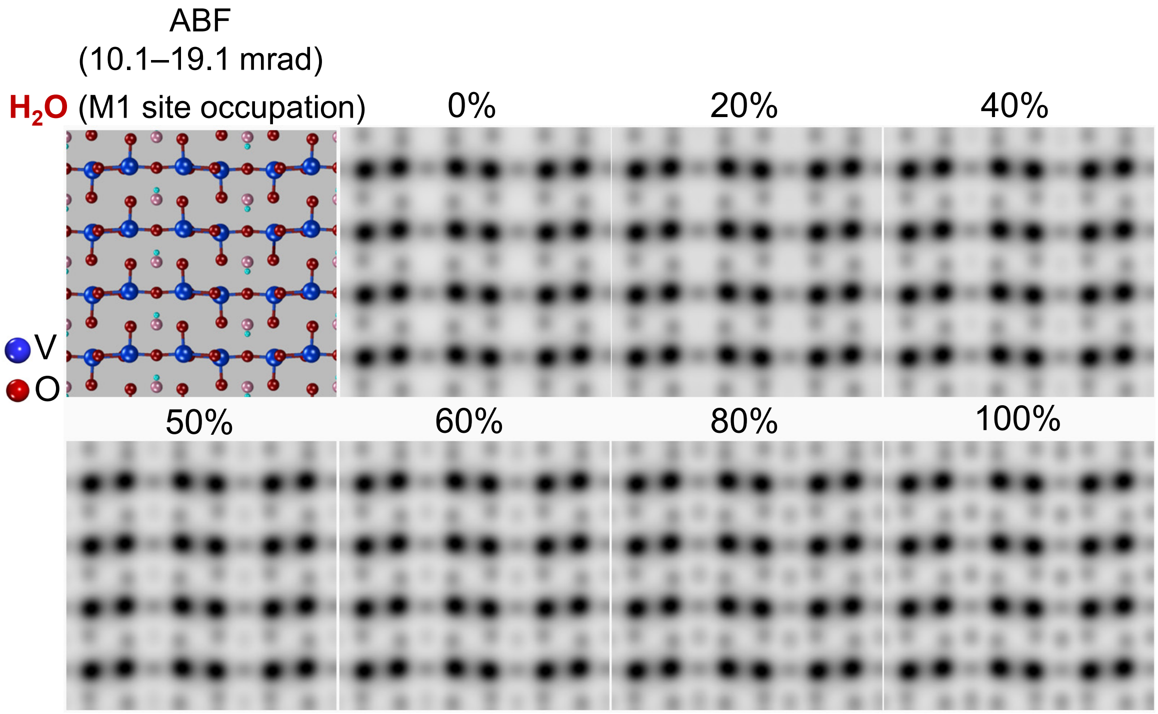

Supplementary Fig. 15 ABF-STEM image simulations with H_2O occupancy at the M1 sites. The proportional increment of the M1-site column contrast by the H_2O occupation is verified. In this thin specimen condition (3 nm), sufficiently visible contrast at the M1 sites is demonstrated when the occupation is ≥50%.

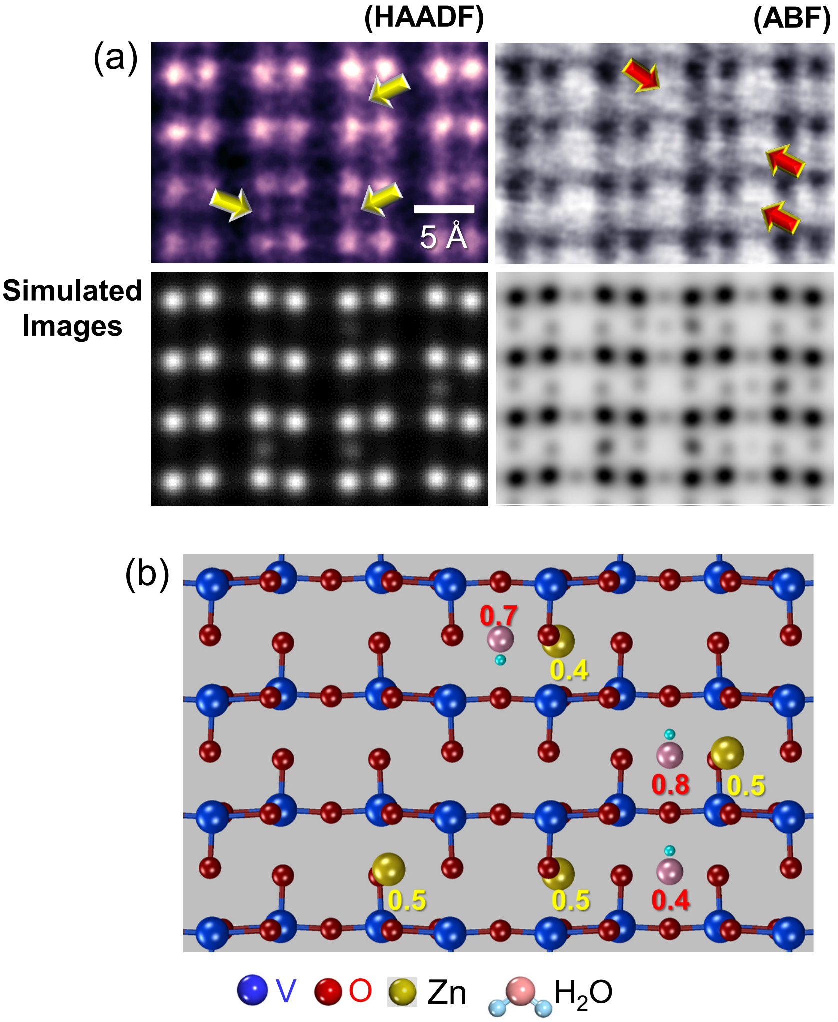

Supplementary Fig. 16 Comparison with simulated images with adequate occupancy factors of Zn and H_2O. (**a**) Good agreement between the real images and the simulated images is noted, verifying the Zn occupation at the M2 sites (yellow arrows) and the H_2O occupation at the M1 sites (red arrows). (**b**) Numeric information of the occupancy factors used for the simulations shown in (a) is provided.

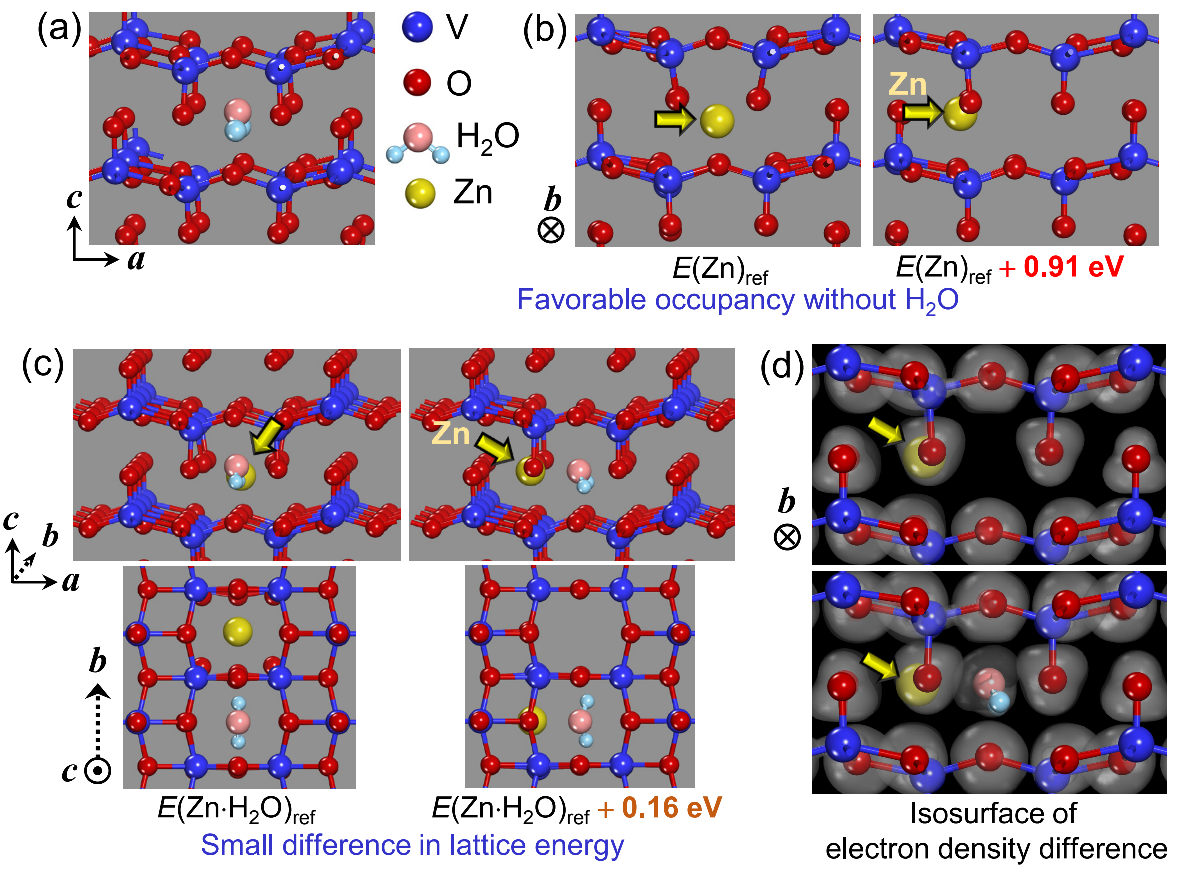

Supplementary Fig. 17 DFT calculations for lattice energy comparison. Yellow arrows indicate Zn atoms. (**a**) The stable configuration of a single H_2O molecule at a M1 site is shown. (**b**) Compared with the M1-site occupancy (left), the pyramidal M2 site Zn occupation (right) has 0.91 eV higher lattice energy, demonstrating that this is energetically unfavorable. (**c**) When a H_2O molecule is inserted, the energy difference between the two occupancies of Zn at the M1 (left) and M2 (right) sites remarkably diminishes to 0.16 eV. (**d**) The isosurface contours of electron-density difference are illustrated with and without a H_2O molecule, respectively. As the H_2O molecule at the M1 site acts as the sixth ligand of Zn, the unusual occupancy of Zn at the M2 site can be stabilized.

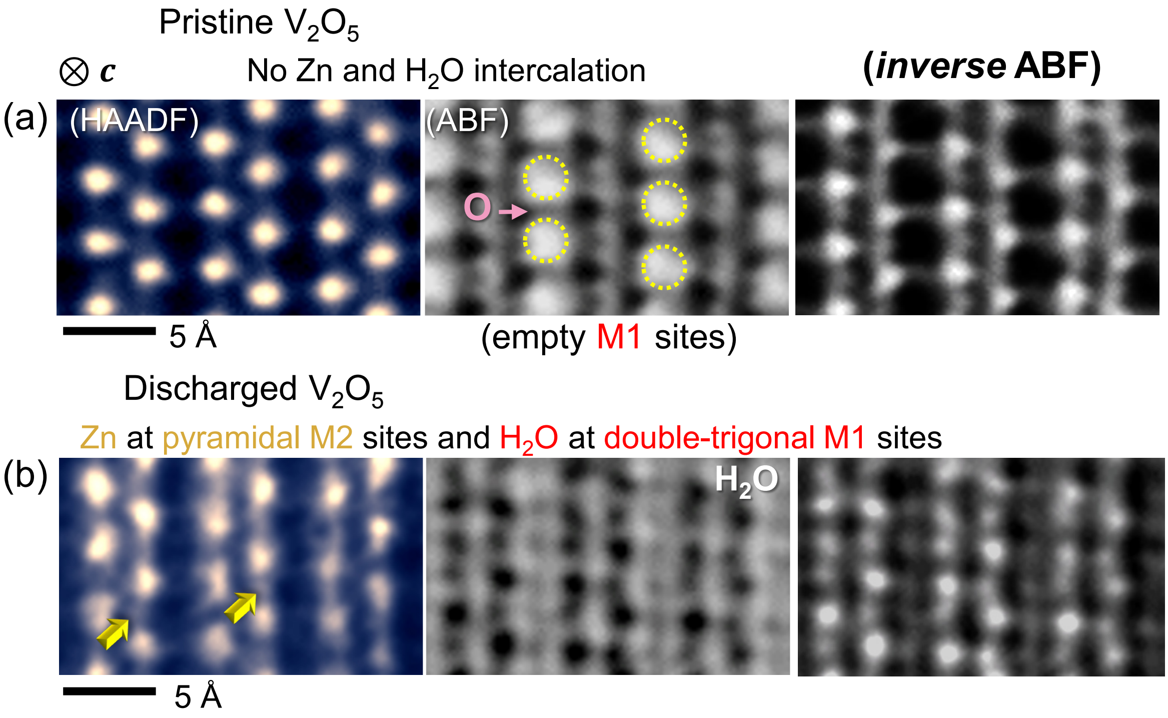

Supplementary Fig. 18 Additional STEM images to visualize the presence of H_2O. (**a**,**b**) These sets of images were acquired in the [001] projection. As denoted by yellow circles in (a), no contrast appears in either HAADF or ABF, indicating that the M1 sites were empty before intercalation. The yellow arrows in the HAADF image in (b) demonstrate the Zn occupancy at the M2 sites. Inverse ABF images with and without H_2O are compared to clarify the H_2O intercalation.

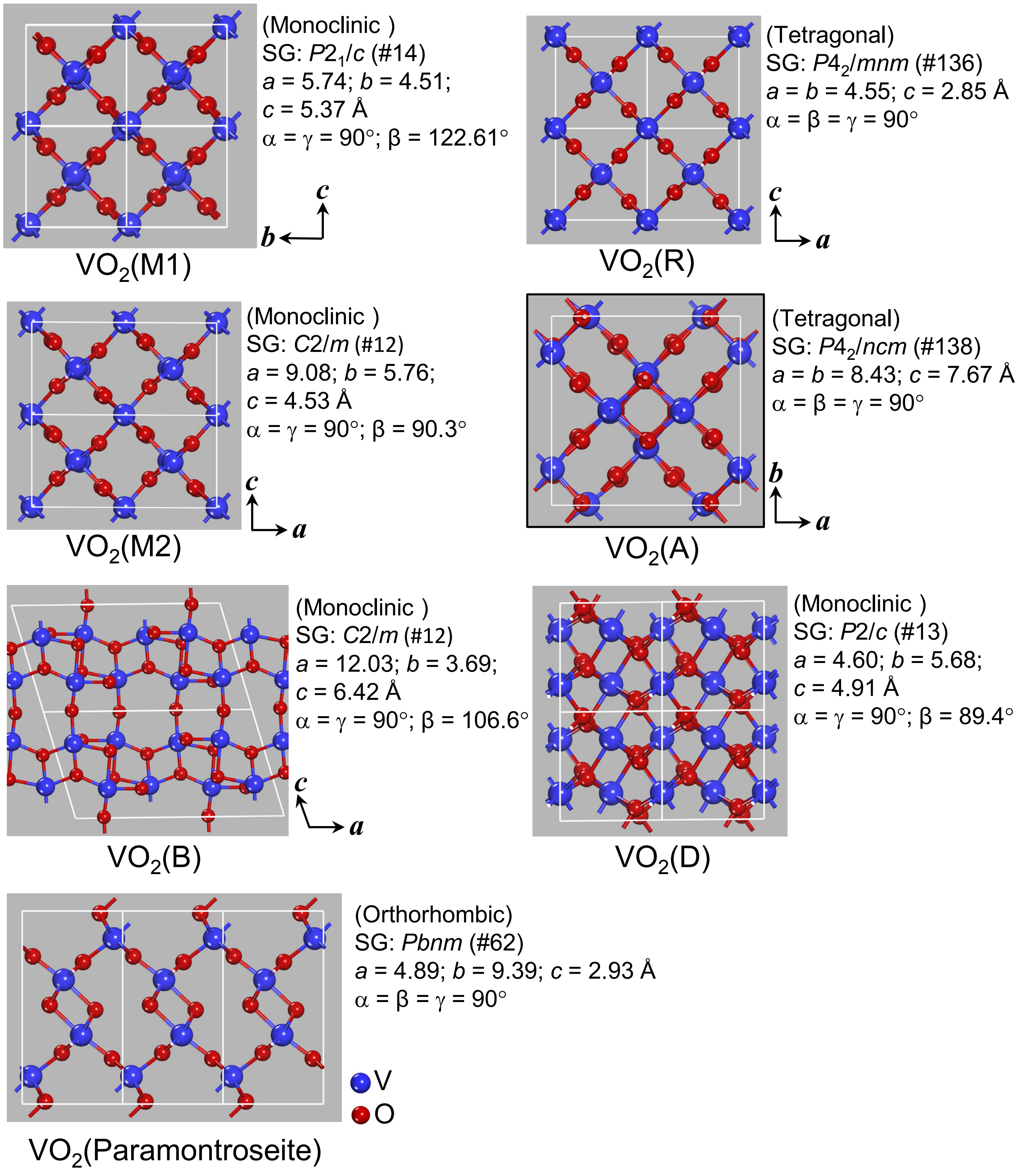

Supplementary Fig. 19 VO_2 Polymorphs. Various polymorphs of VO_2 are shown, demonstrating the distinct crystal structures. In this work, one of the intermediate phases is identified to have the same structure of VO_2(A).

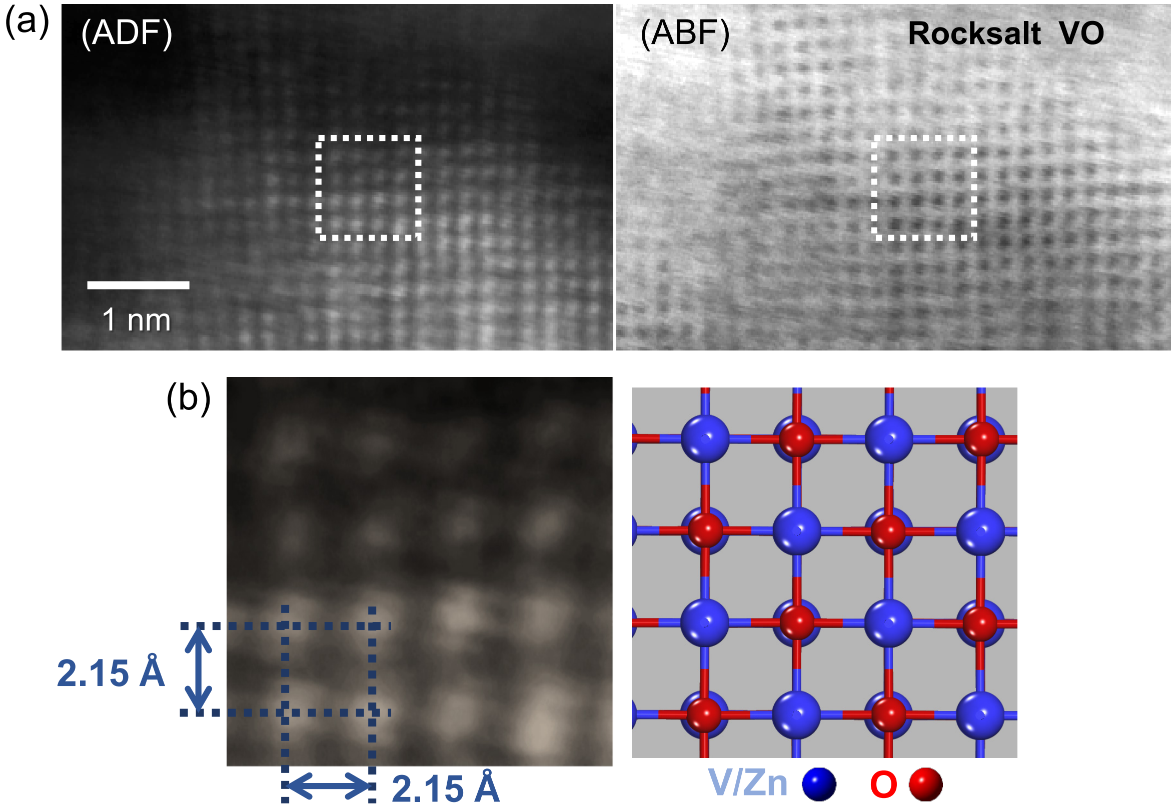

Supplementary Fig. 20 Additional STEM images for the rocksalt VO-type phase. (**a**) A pair of ADF and ABF images clarifies the appearance of the rocksalt phase near a grain boundary having a high Zn concentration. (**b**) A magnified image for the region denoted by a white square in (a) is shown. As indicated on the image, the interplanar distance is measured to be 2.15 Å.

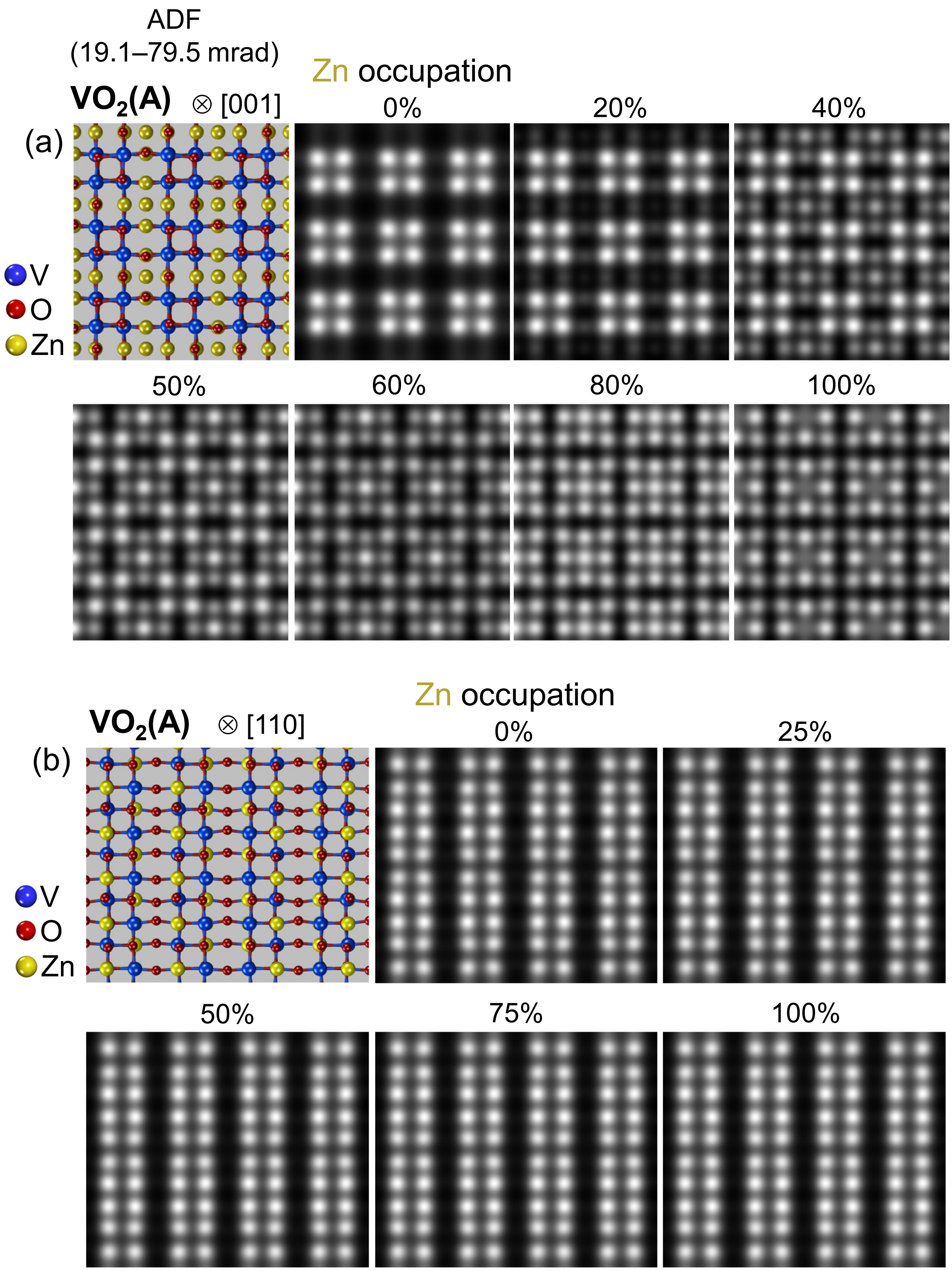

Supplementary Fig. 21 ADF-STEM image simulations of VO_2(A) with Zn occupancy at interstitial sites. Simulations were carried out for two different projections ([001] and [110]). (**a**) The image feature in the [001] projection varies, depending on the degree of Zn occupation. (**b**) In contrast, no substantial variation is recognized in the [110] projection, as the interstitial sites occupied by Zn in this set of simulations overlap with the V columns along the [110] projection.

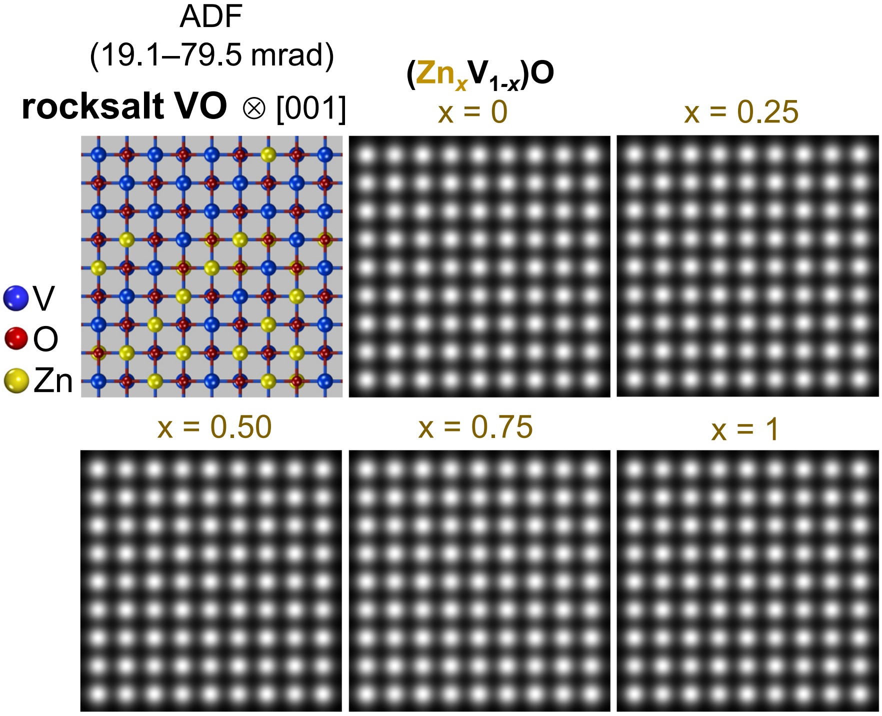

Supplementary Fig. 22 ADF-STEM image simulations of rocksalt VO with Zn. In this series simulations, an influence of Zn substitution at the V sites was examined. As the atomic numbers of V and Zn are fairly similar, the Zn addition as a solid solution hardly changes the image contrast. Simulated images with 40% Zn substitution were used in Figure 5 in the main text.

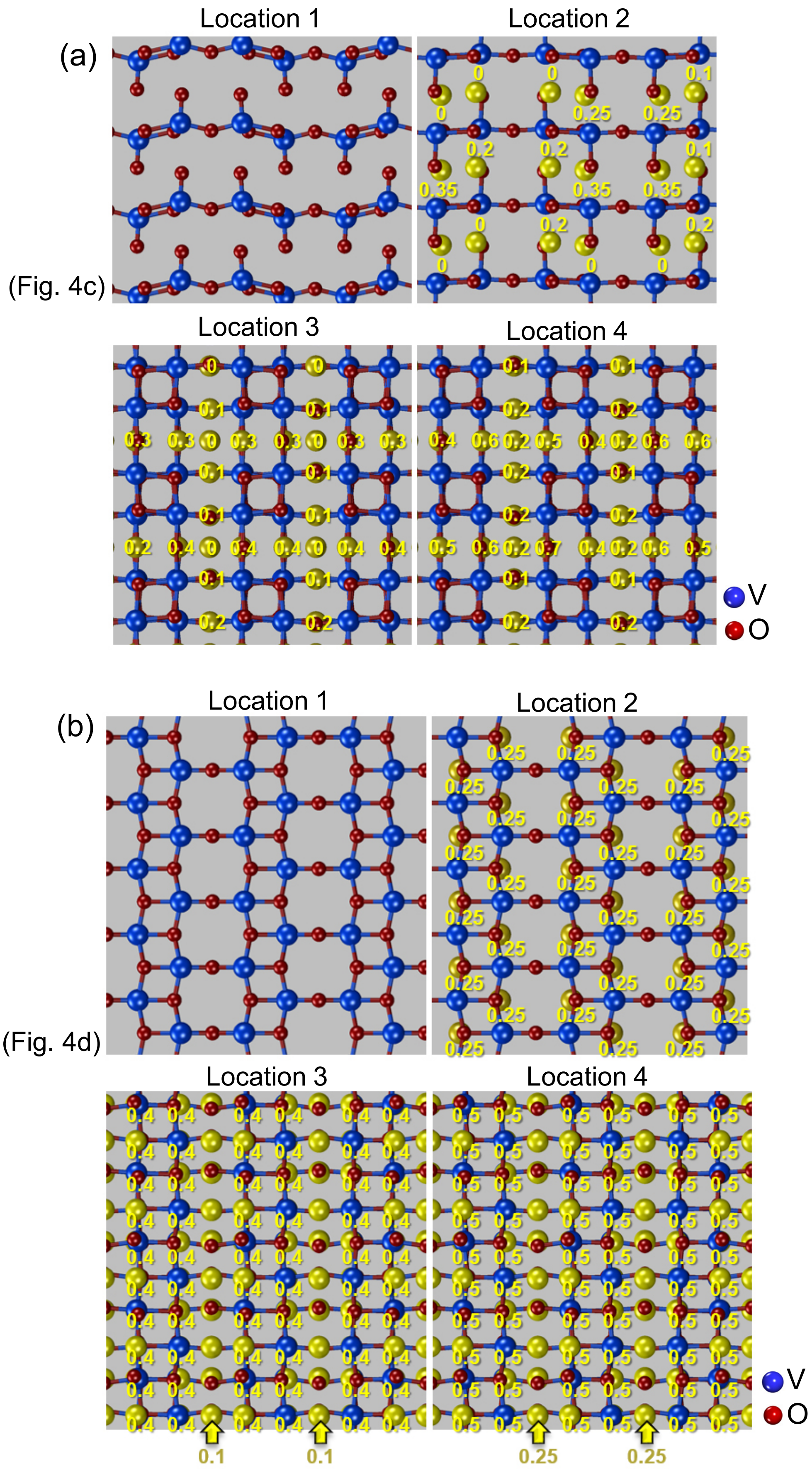

Supplementary Fig. 23 Zn occupation factors used for the image simulations in Figures 5c and 5d. The quantitative information on the occupation factors of Zn to match the real images shown in Figures 5c and 5d in the main text is provided in yellow numbers.

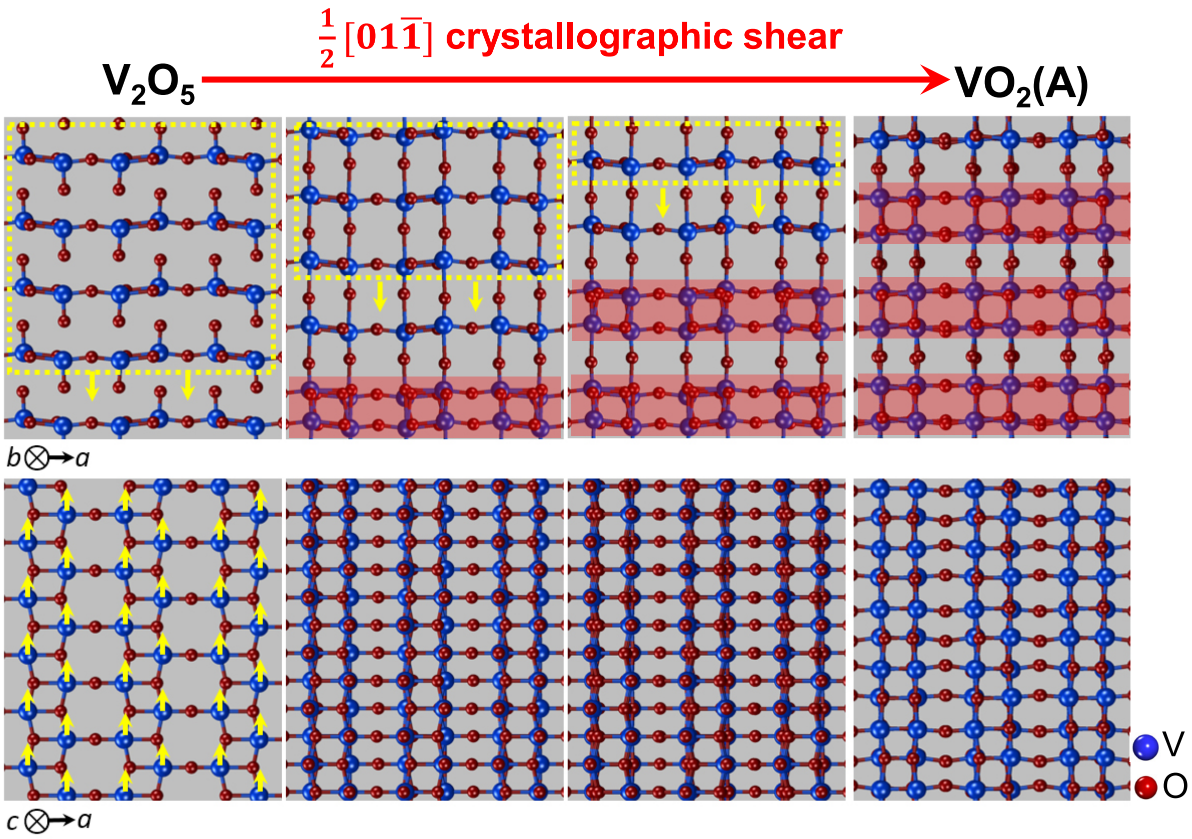

Supplementary Fig. 24 Schematic illustrations for displacements by shearing during the transition from V_2O_5 to VO_2(A). The atomic displacements by massive shearing are schematically depicted by yellow arrows in the two different projections. If this shearing takes place along the [011] direction, the VO_2(A) substructure is constructed in a layer-by-layer manner, as denoted by red shadows.

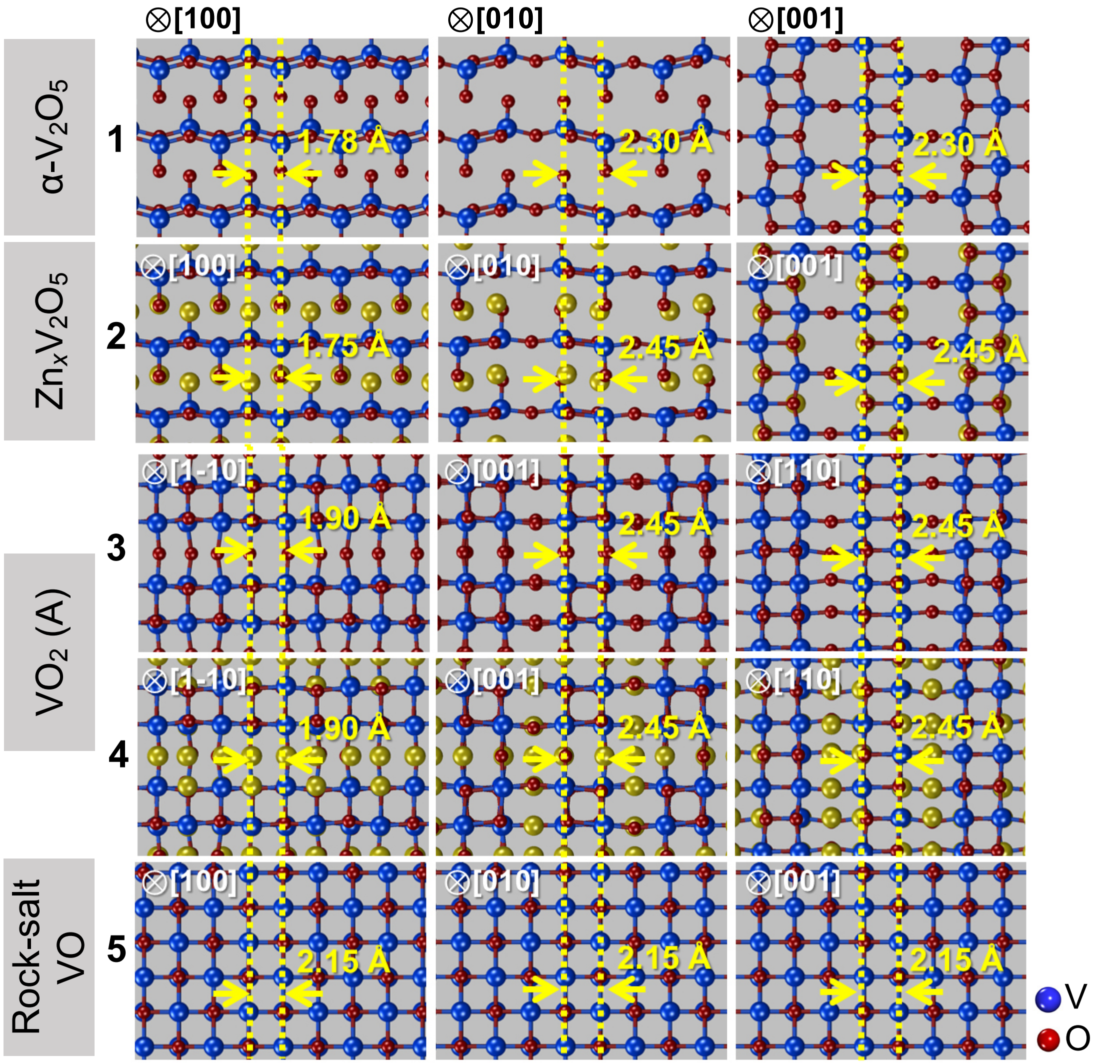

Supplementary Fig. 25 Topotactic structural relationship between the four phases. The atomic arrangements are compared with each other among the four phases (α-V_2O_5, $Zn_xV_2O_5$, VO_2(A), and rocksalt-type VO) in three major zone directions. The comparison of the V−V column distance in the [010] and [001] directions of α-V_2O_5 is made on the basis of direct STEM observations. It is noted that the adjacent V columns along each axis are fairly similar to each other, as noted by yellow numbers and pairs of yellow broken lines. Therefore, this topotactic correlation appears to enable continuous multiphase transformation during the Zn insertion.

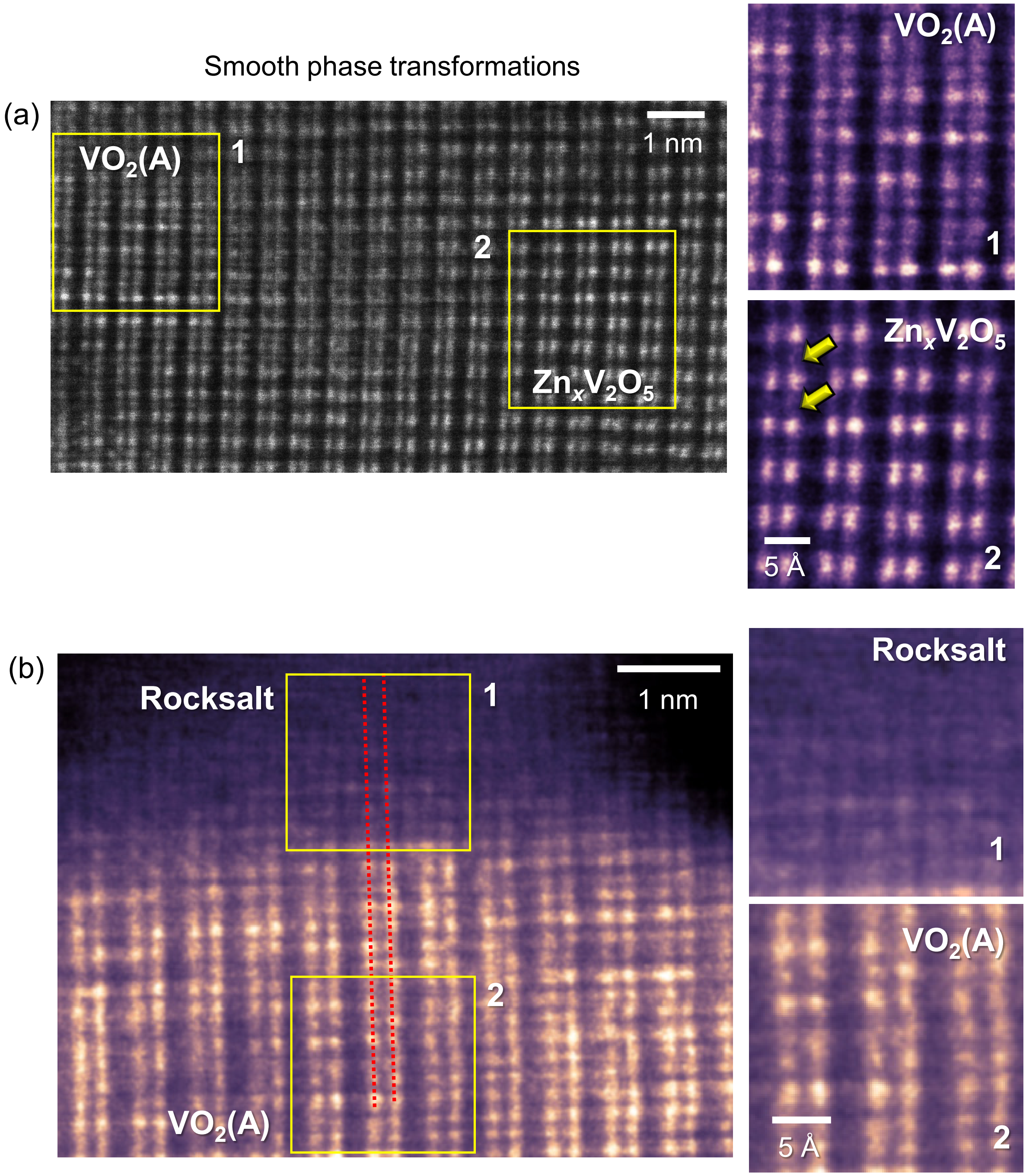

Supplementary Fig. 26 Smooth phase transformations without showing abrupt phase boundaries. (**a**) The ADF image shows the local distribution of the two phases, $Zn_xV_2O_5$ and VO_2(A). It is noted that a clear-cut phase boundary is not identified. As clarified in the magnified images for locations 1 and 2 on the right-hand side, the two different phases are clearly distinguished. Yellow arrows also indicate the bright contrast at the pyramidal M2 sites from the intercalated Zn. (**b**) This image was taken from a grain-boundary region having a high Zn concentration. As denoted by a pair of red lines, a topotatically smooth transformation is also observed between two phases, VO_2(A) (location 2) and rocksalt-type VO (location 1).

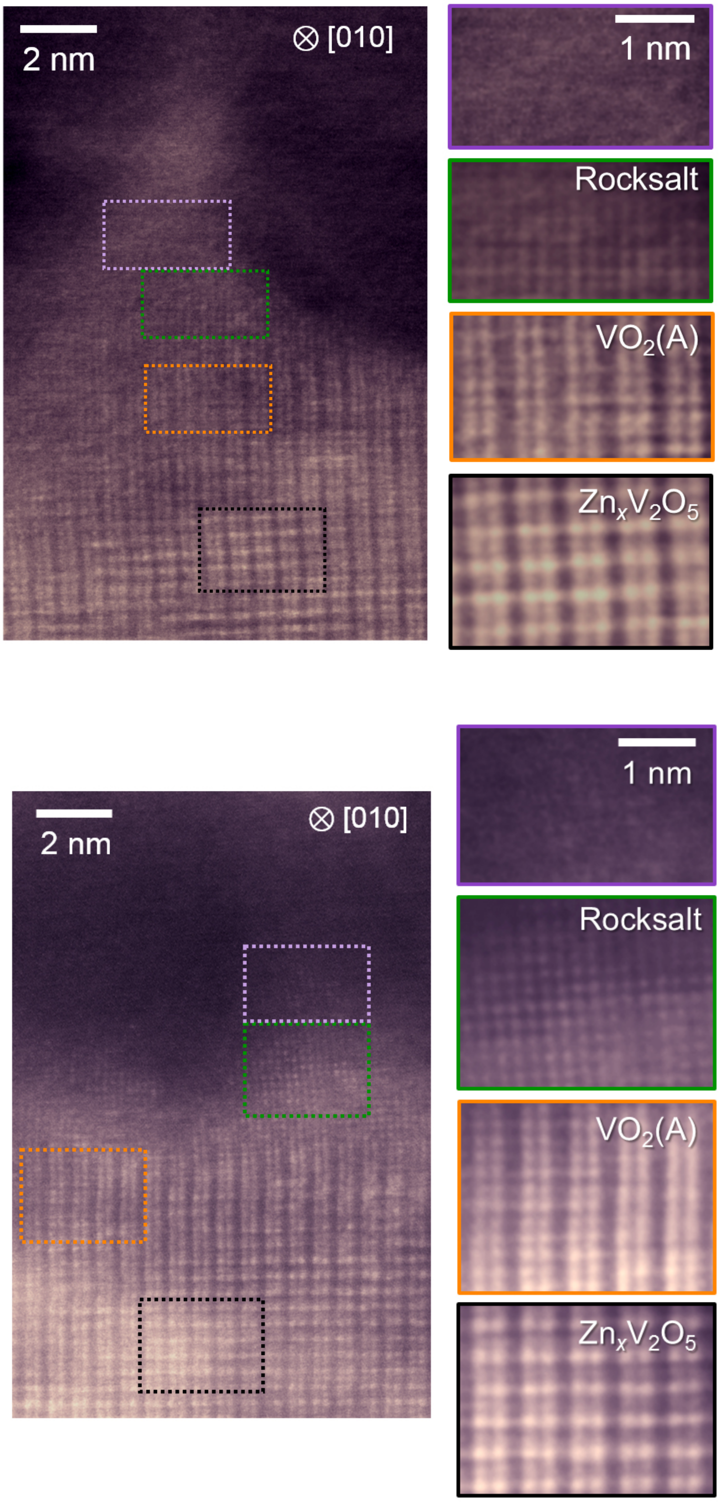

Supplementary Fig. 27 Additional ADF images showing smooth phase transformations. Two independent sets of ADF images are shown together with the enlargements for the local regions denoted by rectangles. These images confirm the structurally smooth and continuous phase transition without showing abrupt lattice discontinuity.

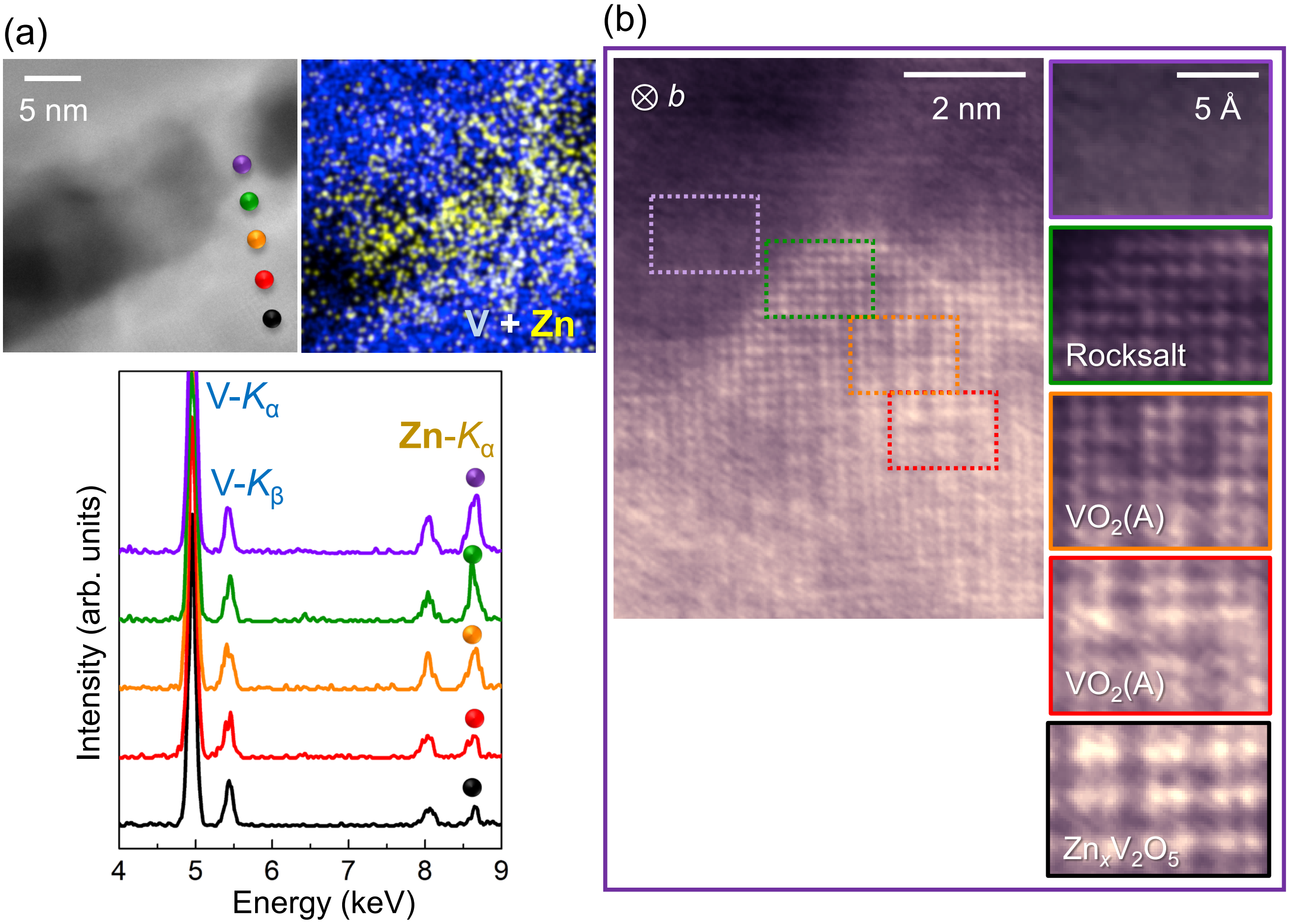

Supplementary Fig. 28 Additional ADF images and EDS chemical information (1). Further STEM and EDS results are provided to verify smooth transformation behavior during Zn insertion. (**a**) As denoted by small spheres in color in the image, consistent variation of Zn concentration near the grain-boundary is shown in a series of EDS spectra along with the compositional map. (**b**) This ADF image and the enlargements for the local regions denoted by rectangles visualize the continuous phase transition.

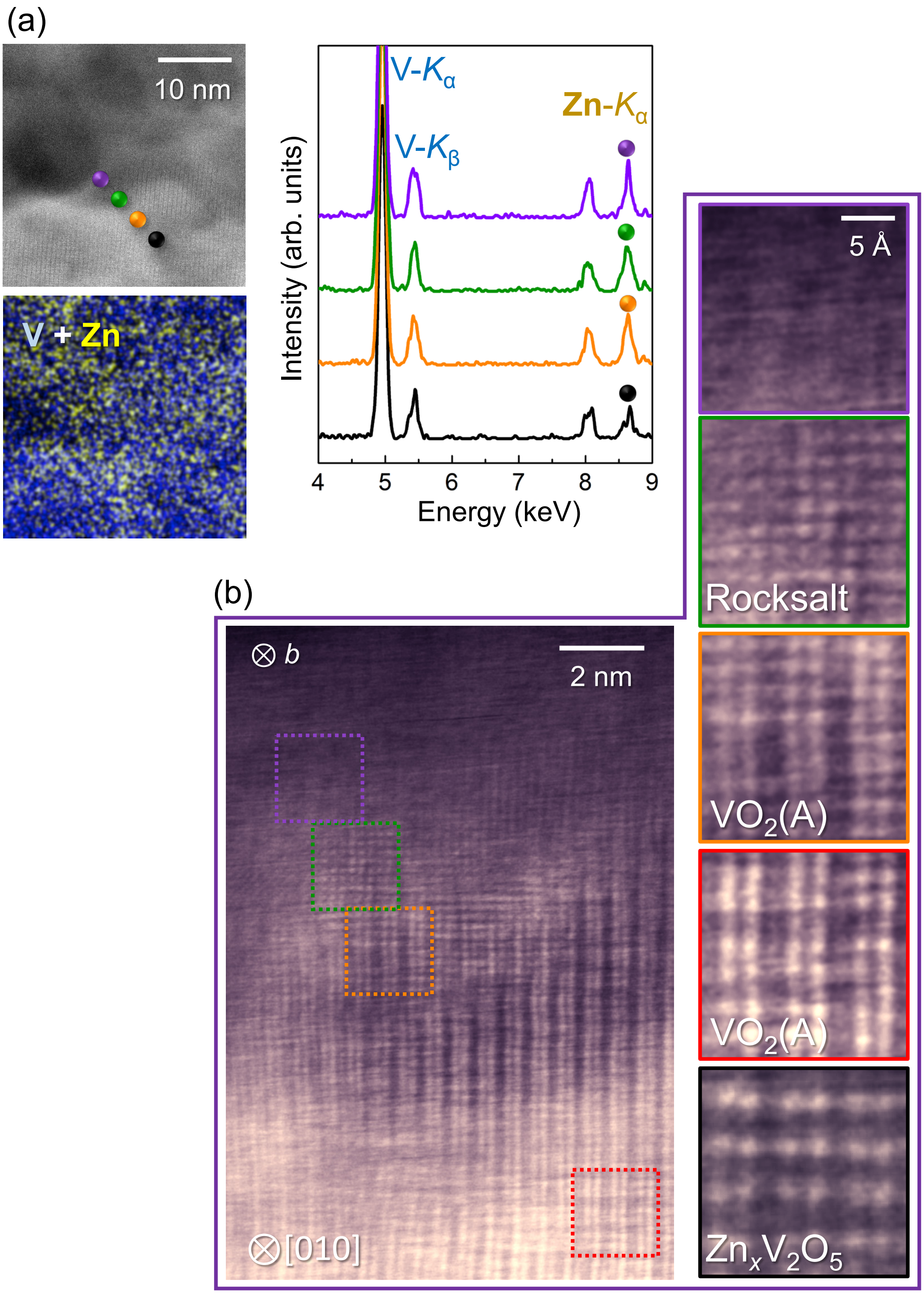

Supplementary Fig. 29 Additional ADF images and EDS chemical information (2). Further STEM and EDS results are provided to verify smooth transformation behavior during Zn insertion. (**a**) Variation of Zn concentration near the grain-boundary is shown in a series of EDS spectra along with the compositional map. (**b**) This ADF image and the enlargements for the local regions denoted by squares visualize the continuous phase transition.

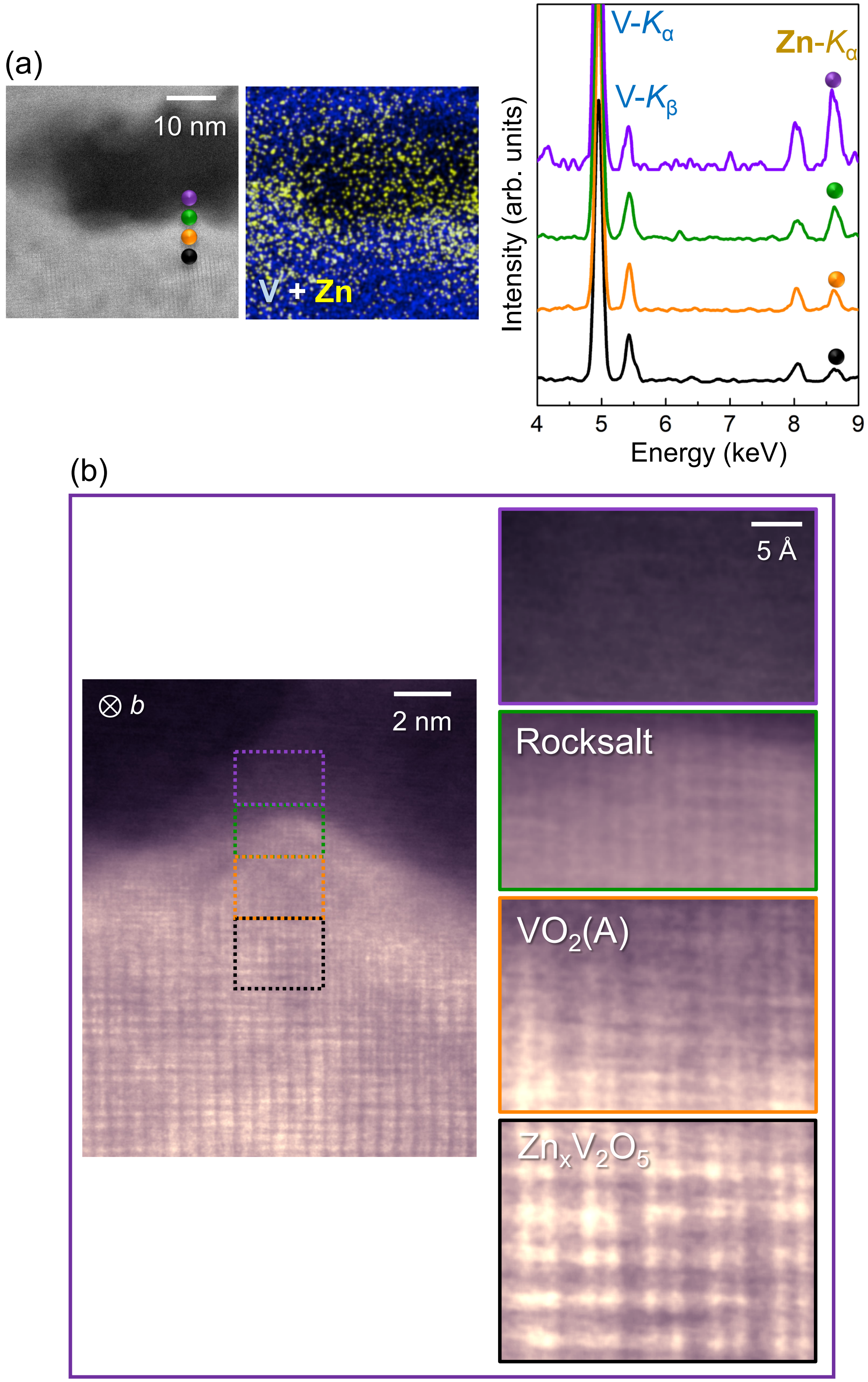

Supplementary Fig. 30 Additional ADF images and EDS chemical information (3). Further STEM and EDS results are provided to verify smooth transformation behavior during Zn insertion. (**a**) Variation of Zn concentration near the grain-boundary is shown in a series of EDS spectra along with the compositional map. (**b**) This ADF image and the enlargements for the local regions denoted by rectangles visualize the continuous phase transition.

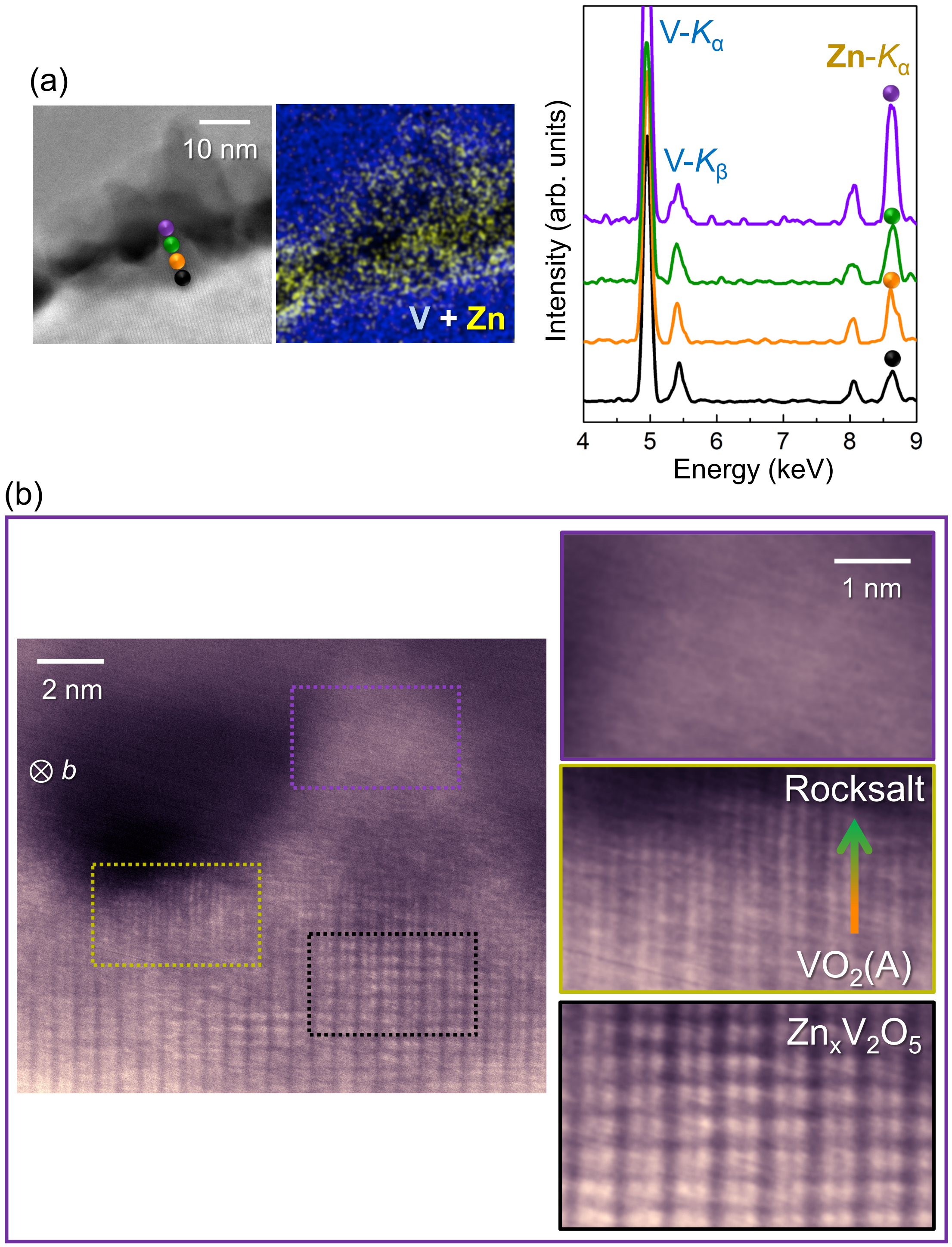

Supplementary Fig. 31 Additional ADF images and EDS chemical information (4). Further STEM and EDS results are provided to verify smooth transformation behavior during Zn insertion. (**a**) Variation of Zn concentration near the grain-boundary is shown in a series of EDS spectra along with the compositional map. (**b**) This ADF image and the enlargements for the local regions denoted by rectangles visualize the continuous phase transition.

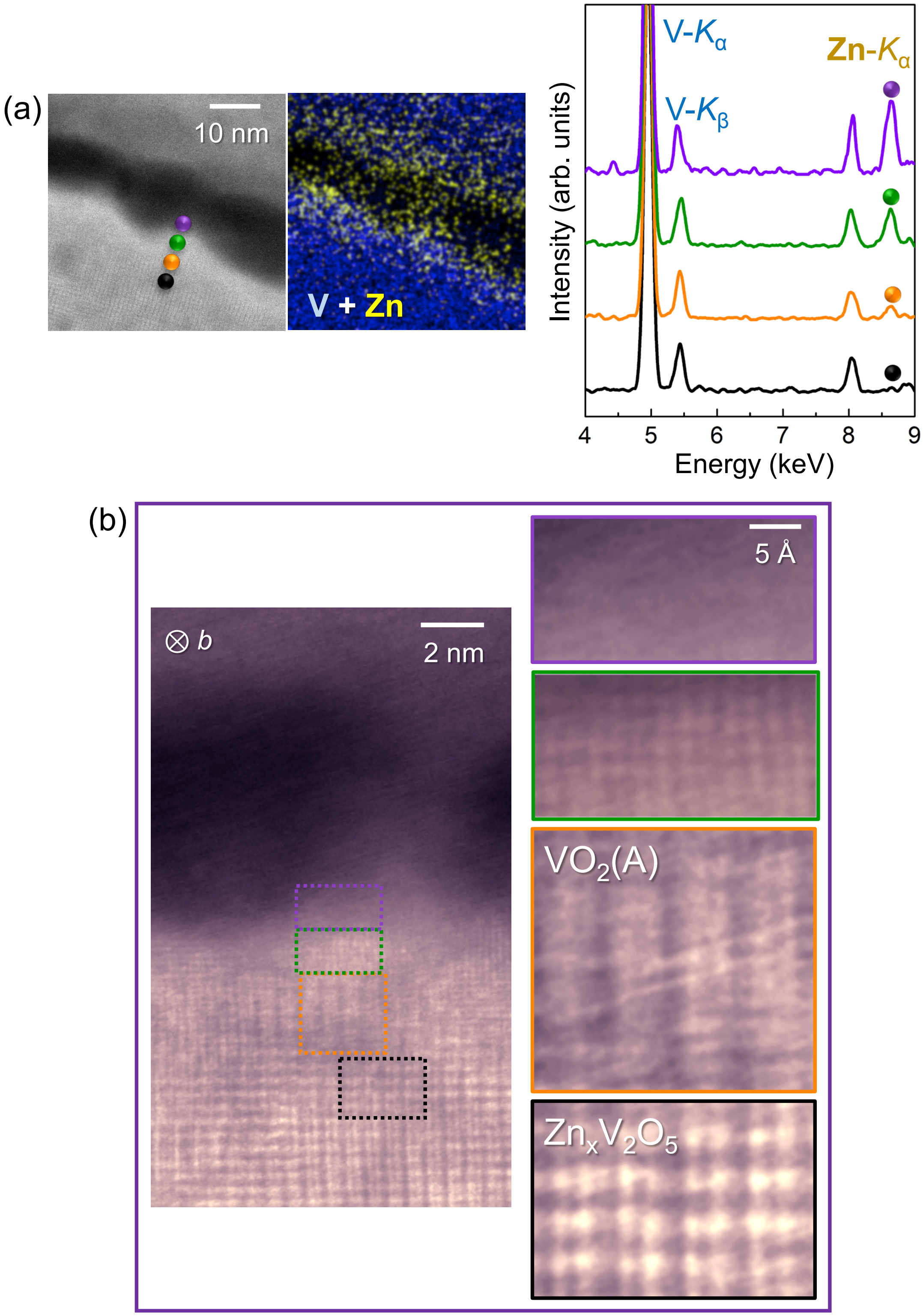

Supplementary Fig. 32 Additional ADF images and EDS chemical information (5). Further STEM and EDS results are provided to verify smooth transformation behavior during Zn insertion. (**a**) Variation of Zn concentration near the grain-boundary is shown in a series of EDS spectra along with the compositional map. (**b**) This ADF image and the enlargements for the local regions denoted by rectangles visualize the continuous phase transition.

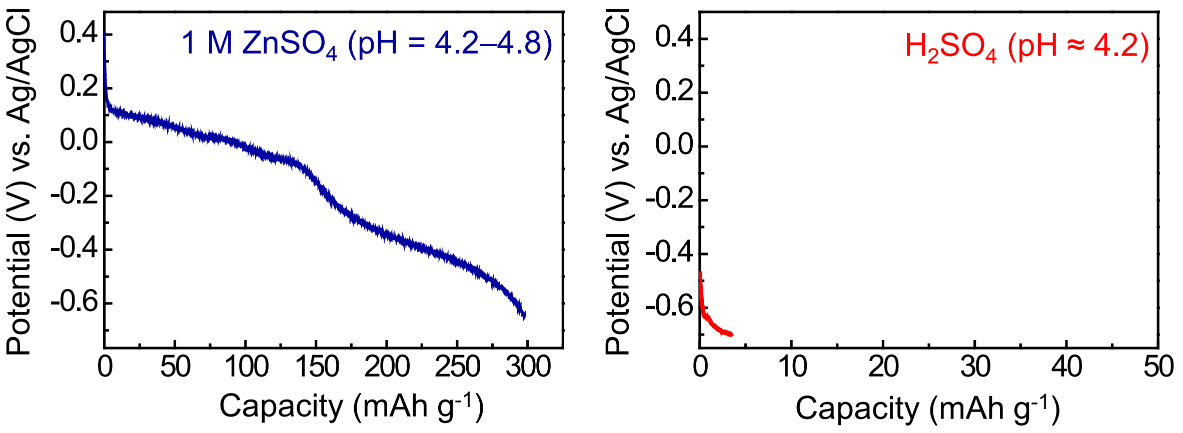

Supplementary Fig. 33 Effect of proton insertion. The pH value of the aqueous electrolytes (1 M $ZnSO_4$) used in this study is in a range of 4.2-4.8. To examine the possibility of proton insertion, the discharge reaction was carried out using a H_2SO_4 solution with pH ≈ 4.2. As directly compared in the two discharge curves, the proton insertion is negligible, showing no substantial capacity. Therefore, all the capacity shown in V_2O_5 is verified to stem from the Zn insertion.

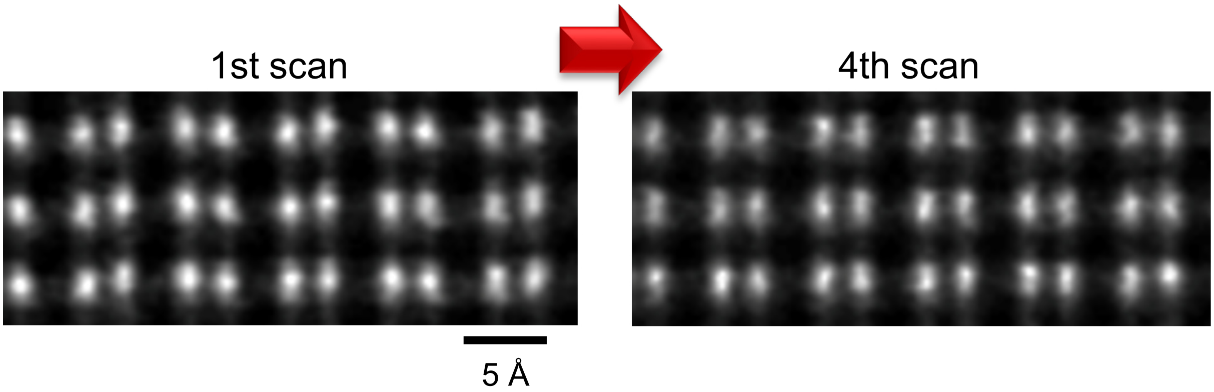

Supplementary Fig. 34 Atomic-column image variation by repetitive e-beam scanning. It is noted that electron-beam-induced contrast degradation in atomic-column imaging is not completely avoidable. As demonstrated, although the V columns become somewhat blurred after the fourth scan during the STEM observation with the beam current of 50 pA, the column resolution in V_2O_5 remains preserved. Therefore, most images for regions of interest in this work were acquired within several scans in STEM.
